# Ultrastrong magnon–magnon coupling dominated by antiresonant interactions

**DOI:** 10.1038/s41467-021-23159-z

**Published:** 2021-05-25

**Authors:** Takuma Makihara, Kenji Hayashida, G. Timothy Noe II, Xinwei Li, Nicolas Marquez Peraca, Xiaoxuan Ma, Zuanming Jin, Wei Ren, Guohong Ma, Ikufumi Katayama, Jun Takeda, Hiroyuki Nojiri, Dmitry Turchinovich, Shixun Cao, Motoaki Bamba, Junichiro Kono

**Affiliations:** 1 Department of Physics and Astronomy, Rice University, Houston, TX USA; 2 Department of Electrical and Computer Engineering, Rice University, Houston, TX USA; 3 Division of Applied Physics, Graduate School of Engineering, Hokkaido University, Sapporo, Japan; 4 Department of Physics, International Center of Quantum and Molecular Structures and Materials Genome Institute, Shanghai University, Shanghai, China; 5 Terahertz Technology Innovation Research Institute, Terahertz Spectrum and Imaging Technology Cooperative Innovation Center, Shanghai Key Lab of Modern Optical System, University of Shanghai for Science and Technology, Shanghai, China; 6 Department of Physics, Graduate School of Engineering Science, Yokohama National University, Yokohama, Japan; 7 Institute for Materials Research, Tohoku University, Sendai, Japan; 8 Fakultät für Physik, Universität Bielefeld, Bielefeld, Germany; 9 Department of Physics I, Kyoto University, Kyoto, Japan; 10 PRESTO, Japan Science and Technology Agency, Saitama, Japan; 11 The Hakubi Center for Advanced Research, Kyoto University, Kyoto, Japan; 12 Department of Materials Science and NanoEngineering, Rice University, Houston, TX USA

**Keywords:** Magnetic properties and materials, Polaritons, Quantum optics

## Abstract

Exotic quantum vacuum phenomena are predicted in cavity quantum electrodynamics systems with ultrastrong light-matter interactions. Their ground states are predicted to be vacuum squeezed states with suppressed quantum fluctuations owing to antiresonant terms in the Hamiltonian. However, such predictions have not been realized because antiresonant interactions are typically negligible compared to resonant interactions in light-matter systems. Here we report an unusual, ultrastrongly coupled matter-matter system of magnons that is analytically described by a unique Hamiltonian in which the relative importance of resonant and antiresonant interactions can be easily tuned and the latter can be made vastly dominant. We found a regime where vacuum Bloch-Siegert shifts, the hallmark of antiresonant interactions, greatly exceed analogous frequency shifts from resonant interactions. Further, we theoretically explored the system’s ground state and calculated up to 5.9 dB of quantum fluctuation suppression. These observations demonstrate that magnonic systems provide an ideal platform for exploring exotic quantum vacuum phenomena predicted in ultrastrongly coupled light-matter systems.

## Abstract (web summary)

Ultrastrong light-matter interactions with dominant antiresonant terms are expected to give rise to interesting phenomena such as quantum fluctuation suppression. Here, the authors propose a system of ultrastrongly coupled magnon modes in a rare earth orthoferrite as a platform for exploring such phenomena.

## Introduction

The interaction of light with solids can exhibit high values of coupling strength, unachievable in atomic and molecular systems, due to large dipole moments and cooperative many-body interactions characteristic of condensed matter. For example, Dicke cooperativity^1^, a quantum optical phenomenon where *N* dipoles coupled to a single electromagnetic field experience a light-matter coupling strength enhanced by a factor of $\sqrt{N}$, becomes drastic in condensed matter. Leveraging cooperative many-body interactions enables observations of the exotic ultrastrong coupling (USC) regime^2,3^.

In the USC regime, the light-matter coupling strength becomes comparable to the bare frequencies of the system. In this regime, the rotating-wave approximation (RWA) breaks down, leading to antiresonant interactions from the so-called counter-rotating terms (CRTs) and *A*^2^ terms in the Hamiltonian, which allow access to counter-intuitive and unexplored physics. In the past decade, USC has been realized in diverse physical platforms, including intersubband polaritons^4,5^, Landau polaritons^6–8^, and superconducting circuits^9–12^. However, traditional polariton systems are restricted by one fixed coupling strength, and resonant effects, such as vacuum Rabi splitting (VRS), dominate antiresonant effects, such as vacuum Bloch–Siegert shifts (VBSSs)^13^, which are the hallmark of active CRTs. Thus, the counter-intuitive physics predicted in this regime, such as the superradiant phase transition^14,15^, Casimir photon emission^16–18^, and ground-state electroluminescence^19^, has largely remained unexplored. Experimental studies are largely limited to reports of giant VRS, and there have only been a few unambiguous demonstrations of the VBSS^20^. Therefore, there is a growing demand for platforms with superior tunability and dominant antiresonant interactions for exploring the exotic predictions of the USC regime.

Here, we demonstrate matter-matter USC in YFeO_3_, a rare-earth orthoferrite^21^, that is analytically described by a unique cavity quantum electrodynamics (QED) Hamiltonian with tunable coupling strengths and dominant counter-rotating interactions. We systematically examined how the quasi-ferromagnetic (qFM) and quasi-antiferromagnetic (qAFM) magnon modes modes interact with each other by characterizing their resonance frequencies at different applied magnetic field strengths and directions. We were able to use the applied magnetic field to tune the VRS and VBSSs, and in certain geometries, the frequency shifts of the coupled modes were dominated by the VBSSs and not the vacuum Rabi splitting-induced shifts (VRSSs). A well-established microscopic spin model of this material system^22^ accurately reproduced our observed resonances without any adjustable parameters. We show that this lightless spin model can be precisely mapped to a polariton model described by an anisotropic Hopfield Hamiltonian in which the magnon–magnon coupling strengths are easily tunable and the CRTs dominate the co-rotating terms, consistent with our observation of giant VBSSs. Finally, we theoretically investigated the ground state of our system and demonstrate that it is intrinsically squeezed, consisting of a two-mode squeezed vacuum as expected in the USC regime^23–25^, with quantum fluctuation suppression as large as 5.9 dB.

## Results

### Terahertz time-domain magnetospectroscopy

**Fig. 1:**
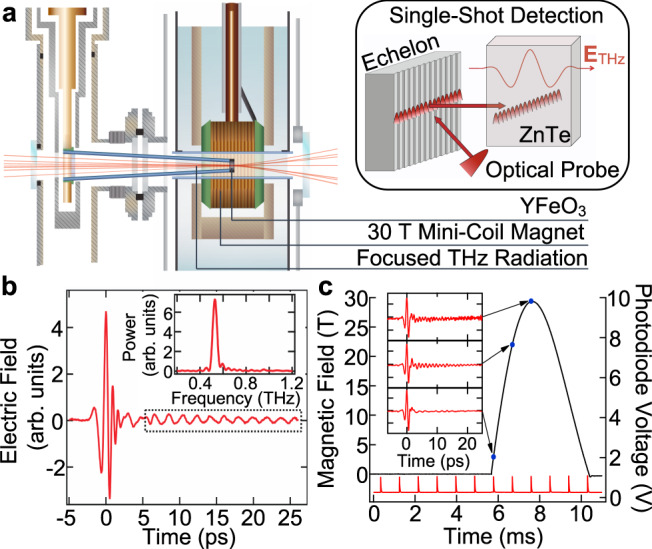
Unique combination of table-top, 30 T pulsed magnet and single-shot THz detection. **a** Schematic of pulsed magnet surrounding single crystals of YFeO_3_. Note that although the sample is held on a sapphire pipe mounted on the cold finger of a liquid helium cryostat, no liquid helium was used in this study. Single-shot detection (shown in inset) is based on a combination of a zinc telluride (ZnTe) crystal and a unique reflective echelon. **b** Sample THz electric field waveform transmitted through a YFeO_3_ crystal. Time-domain oscillations for *t* > 0 from coherent spin precessions (magnons) are Fourier transformed to yield the magnon frequency (inset). **c** Pulsed magnetic field profile (solid black line), optical pulses used to generate/detect THz waveforms (solid red line), and sampled magnetic field strengths (blue dots) with transmitted THz waveforms measured at the sampled magnetic field strength shown in the inset. The optical pulses are detected using a photodiode that measures scattered light.

To interrogate magnons in YFeO_3_, we used terahertz time-domain spectroscopy (THz TDS). In THz TDS studies of rare-earth orthoferrites, free-induction decay signals from precessing spins are measured directly in the time domain, the Fourier transform of which reveal the precessional (magnon) frequency^26^. We combined two unique experimental apparatuses: a table-top, 30 T pulsed magnet^27^ and single-shot THz detection^28,29^, illustrated in Fig. 1a. THz pulses were focused onto the samples, and the transmitted THz waveform was detected using a single-shot technique based on a reflective echelon that separates an optical probe pulse into time-delayed beamlets that overlap with the THz waveform in our ZnTe detection crystal^28,29^. Figure 1b displays a THz waveform transmitted through YFeO_3_ and detected using single-shot detection. Coherent oscillations are observed for *t* > 0, whose Fourier transform reveals the magnon frequency (inset). Figure 1c shows the magnetic field profile, the detection optical pulses, and the sampled magnetic field strengths, as well as the THz waveforms measured at the sampled field strengths.

### Demonstration of ultrastrong magnon–magnon coupling

**Fig. 2:**
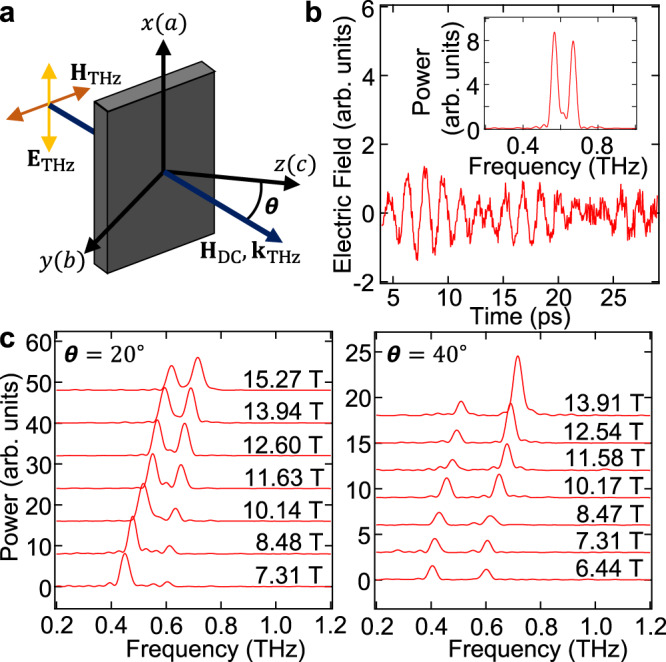
Magnon signals in time and frequency domains. **a** Schematic of THz magnetospectroscopy studies of YFeO_3_ in a tilted magnetic field. **H**_DC_ was applied in the *b−c* plane at an angle of *θ* with respect to the *c*-axis, with **k**_THz_//**H**_DC_ and **H**_THz_ polarized in the *b*−*c* plane. **b** Transmitted THz waveform for *θ* = 20^∘^ at **H**_DC_ = 12.60 T displaying beating in the time-domain and two peaks in the frequency domain corresponding to the simultaneous excitation of both magnon modes in YFeO_3_. **c** Magnon power spectra for *θ* = 20^∘^ and *θ* = 40^∘^ at different **H**_DC_ displaying larger frequency splitting for larger *θ*.

In the absence of an applied external magnetic field (**H**_DC_ = 0), YFeO_3_ crystallizes in an orthorhombic perovskite structure. Its magnetic structure is described by the Γ_4_ phase, where the two Fe^3+^ spin sublattices (**S**_1_ and **S**_2_) order antiferromagnetically along the *a*-axis, with a slight canting towards the *c*-axis due to the Dzyaloshinskii–Moriya interaction. Figure 2 shows results of THz magnetospectroscopy studies of YFeO_3_. We studied five different single crystals of YFeO_3_ cut such that the applied magnetic field, **H**_DC_, was directed at angles of *θ* = 0^∘^, 20^∘^, 40^∘^, 60^∘^, and 90^∘^ with respect to the *c*-axis in the *b-c* plane. The measurements were conducted at room temperature in the geometry shown in Fig. 2a. The THz radiation propagated parallel to **H**_DC_, and the incident THz electric field **E**_THz_ was linearly polarized along the *a*-axis. In general, the emitted THz electric fields were elliptically polarized^30^, so THz electric fields polarized parallel to the *a*-axis (${{\bf{E}}}_{{\rm{THz}}}^{a}$) and polarized in the *b-c* plane (${{\bf{E}}}_{{\rm{THz}}}^{b-c}$) were both measured to fully characterize the magnetic resonances^26,30^.

**Fig. 3:**
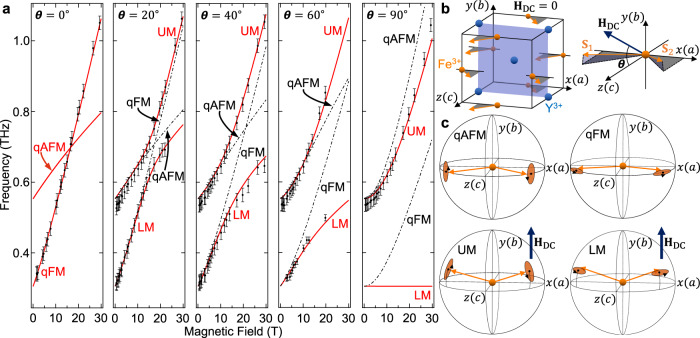
Evidence for dominant vacuum Bloch-Siegert shifts. **a** Experimentally measured magnon frequencies for *θ* = 0^∘^, 20^∘^, 40^∘^, 60^∘^, 90^∘^ versus **H**_DC_ (black dots) with calculated resonance magnon frequencies (solid red lines) and decoupled quasi-ferromagnetic (qFM) and quasi-antiferromagnetic (qAFM) magnon frequencies (black dashed-dotted lines). The upper mode (UM) frequency becomes lower than the qAFM frequency at *θ* = 90^∘^, indicating a dominant VBSS compared to the VRSS. Error bars are 1/*T* where *T* is the Fourier-transformed time-window and is limited by our THz detection. **b** Crystal and magnetic structure of YFeO_3_. **c** Spin dynamics in the decoupled qFM and qAFM modes, as well as the UM and lower mode (LM).

Figure 2b displays an example THz waveform and its Fourier transform for an applied field strength of 12.60 T at *θ* = 20^∘^. Beating in the time domain, and correspondingly two peaks in the frequency domain, indicate the simultaneous excitation of two magnon modes. Figure 2c displays an example of the two magnon frequencies extracted by Fourier transforming ${{\bf{E}}}_{{\rm{THz}}}^{b{\rm{-}}c}$ for *θ* = 20^∘^ and 40^∘^ at different magnetic fields (see Methods for all measurements). Figure 3a plots the observed resonance frequencies (black dots) versus magnetic field for all measured *θ*. We observe anticrossing between the two frequencies whose splitting increases with increasing *θ*, illustrating strong coupling between the two magnons with tunable coupling strengths. Further, the frequency splitting is comparable to the bare magnon frequencies, indicating ultrastrong magnon–magnon coupling.

We first interpret our observed magnon–magnon coupling by considering the symmetry of the spin dynamics in the qFM and qAFM modes. Figure 3b displays the crystal and magnetic structure of YFeO_3_. Figure 3c qualitatively illustrates the spin precessions in the qFM and qAFM modes for **H**_DC_ = 0. In this geometry, or when **H**_DC_ is applied along the *c*-axis (*θ* = 0^∘^), **S**_1_ and **S**_2_ maintain *π* rotational symmetry about the *c*-axis, and the qFM and qAFM modes do not hybridize due to opposite parities under this symmetry: the qAFM mode is unchanged whereas the qFM mode gains a *π* phase shift^31,32^. However, this symmetry is broken when the spins possess a component along the *b*-axis, allowing the qFM and qAFM modes to hybridize. We employ a tilted **H**_DC_ in the *b-c* plane to prepare an equilibrium spin configuration where **S**_1_ and **S**_2_ posses components along the *b*-axis, as in Fig. 3b, and enable hybridization.

### Microscopic spin model

To quantitatively illustrate the hybridization between the qFM and qAFM modes, we numerically analyzed the spin dynamics in a tilted magnetic field. We started from a microscopic spin model describing interactions between **S**_1_ and **S**_2_, including the symmetric exchange, the antisymmetric exchange, the single-ion anisotropies, and the Zeeman interaction^22^. The model contains no fitting parameters; the inputted magnetic parameters are well-known for YFeO_3_^33^. By solving the Landau–Lifshitz–Gilbert equation, we obtained the spin dynamics in a tilted magnetic field. Figure 3a plots the resonance frequencies (solid red lines), which excellently reproduce our experimental results (black dots). We observe that for nonzero *θ*, the resonance frequencies display anticrossing, indicating mode hybridization consistent with the broken *π* rotational symmetry mentioned above. The two coupled modes are labeled as the upper mode (UM) and lower mode (LM) for the higher and lower frequency branches, respectively. We do not excite the qAFM mode when *θ* = 0^∘^ and we do not excite the LM when *θ* = 90^∘^ due to magnon excitation selection rules (see Methods).

We calculated the dynamics of the decoupled qFM and qAFM modes in a tilted magnetic field, which are uniquely defined by opposite parities under *π* rotation about the *c*-axis, by neglecting coupling between these independent spin precessions in the equations of motion. The decoupled magnon frequencies are plotted as black dashed-dotted lines in Fig. 3a for nonzero *θ*. Note that for *θ* = 0^∘^, the qFM and qAFM modes solve the full equations of motion. For *θ* = 20^∘^, 40^∘^, and 60^∘^, we observe that the UM is higher in frequency than the qFM and qAFM modes. This is precisely what one expects for hybridization within the RWA; for the UM, the VRSS (exclusively from the co-rotating interaction) is always a blue-shift. However, we observe that for *θ* = 90^∘^, the UM is lower in frequency than the qAFM mode, indicating an additional red-shift of the coupled magnon frequencies. This dominant red-shift, which is a direct consequence of the counter-rotating term, is the dominant VBSS. Due to giant VBSSs, we observe that the UM and LM, whose dynamics are qualitatively illustrated in Fig. 3c for *θ* = 90^∘^, not only hybridize the qFM and qAFM modes but also contain the time-reversed dynamics of the qFM and qAFM modes (see Methods). Additional calculations showing the transition from *θ* = 60^∘^ to *θ* = 90^∘^ are shown in Supplementary Fig. 11.

### Quantum mechanical model

To evaluate the magnon–magnon coupling strengths, we rewrite our microscopic spin model in terms of the creation and annihilation operators of the qFM and qAFM magnons:

1 $${\mathcal{H}}=	\; \hslash {\omega }_{0a}\left({\hat{a}}^{\dagger }\hat{a}+\frac{1}{2}\right)+\hslash {\omega }_{0b}\left({\hat{b}}^{\dagger }\hat{b}+\frac{1}{2}\right)+i\hslash {g}_{1}\left(\hat{a}{\hat{b}}^{\dagger }-{\hat{a}}^{\dagger }\hat{b}\right)\\ 	 +i\hslash {g}_{2}\left({\hat{a}}^{\dagger }{\hat{b}}^{\dagger }-\hat{a}\hat{b}\right),$$

where $\hat{a}$ (${\hat{a}}^{\dagger }$) annihilates (creates) a qFM magnon with frequency *ω*_0*a*_, and $\hat{b}$ (${\hat{b}}^{\dagger }$) annihilates (creates) a qAFM magnon with frequency *ω*_0*b*_, where *ω*_0*a*_ and *ω*_0*b*_ are the frequencies of the decoupled qFM and qAFM modes discussed in the previous paragraph. Expressions for the co-rotating coupling strength (*g*_1_) and the counter-rotating coupling strength (*g*_2_), which are derived in the absence of adjustable parameters, are provided in the Methods. Our Hamiltonian resembles the Hopfield Hamiltonian^34^, which is related to the paradigmatic Dicke Hamiltonian by a Holstein–Primakoff transformation. However, unlike the Hopfield Hamiltonian and analogous light-matter Hamiltonians, such as the quantum Rabi model or the Dicke Hamiltonian, our system is not restricted to *g*_1_ = *g*_2_. Although similar anisotropic Hamiltonians, such as the anisotropic quantum Rabi model, have been theoretically proposed^35^ and experimentally realized in superconducting circuits^36^, our condensed matter system can simulate many-body Hamiltonians. For example, the Hopfield Hamiltonian is typically used in studies of USC in condensed matter systems, such as intersubband polaritons^25^ and exciton-polaritons^37,38^.

**Fig. 4:**
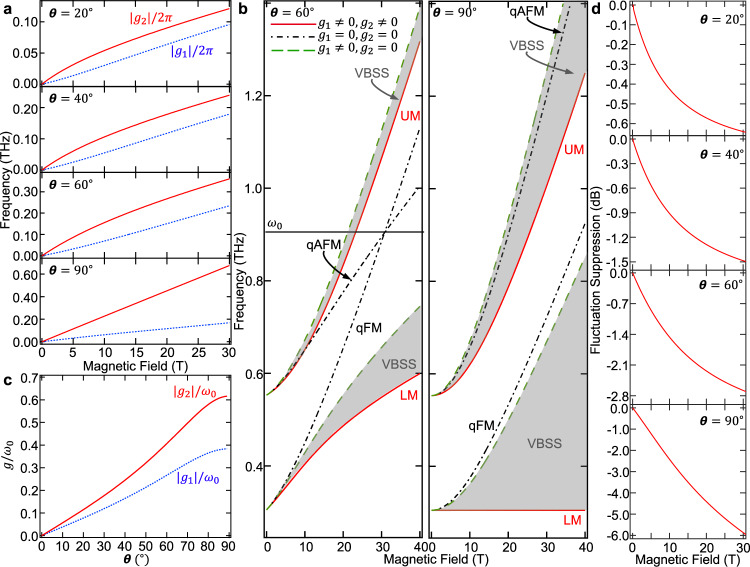
Extreme breakdown of rotating-wave approximation and vacuum squeezing. **a** Co-rotating (∣*g*_1_∣/2*π*, blue dotted line) and counter-rotating (∣*g*_2_∣/2*π*, red solid line) coupling strengths for *θ* = 20^∘^, 40^∘^, 60^∘^, 90^∘^ displaying dominance of the counter-rotating term. **b** Theoretical illustration of the quasi-ferromagnetic (qFM) mode, quasi-antiferromagnetic (qAFM) mode, lower mode (LM), upper mode (UM), and co-rotating coupled magnon frequencies that are obtained by setting *g*_2_ = 0, for *θ* = 60^∘^ and 90^∘^. The vacuum Bloch–Siegert shifts (VBSSs) are highlighted by the shaded area. **c** Normalized co-rotating (∣*g*_1_∣/*ω*_0_, blue dotted line) and counter-rotating (∣*g*_2_∣/*ω*_0_, red solid line) coupling strengths displaying ultrastrong magnon–magnon coupling and dominance of the counter-rotating terms (CRTs). *ω*_0_ is the frequency at which the qFM and qAFM modes cross, as illustrated in **b**. **d** Fluctuation suppression in ${\hat{X}}_{\hat{c},\phi }$ evaluated in the ground state of the coupled magnon system for *θ* = 20^∘^, 40^∘^, 60^∘^, and 90^∘^ demonstrating squeezing. For *θ* = 90^∘^, suppression reaches 5.9 dB for 30 T.

Figure 4a plots values of ∣*g*_1_∣ and ∣*g*_2_∣ versus applied magnetic field for *θ* = 20^∘^, 40^∘^, 60^∘^, and 90^∘^ (see Supplementary Fig. 13 for plots of ∣*g*_1_∣ and ∣*g*_2_∣ versus *θ* for different applied magnetic fields). We find that *g*_1_ and *g*_2_ exactly vanish when the applied magnetic field vanishes and when *θ* = 0^∘^ (not shown). Importantly, Fig. 4a demonstrates tunable, anisotropic co-rotating and counter-rotating coupling strengths, with the latter always dominating the former, indicating an extreme breakdown of the RWA. This observation builds on recent experiments^39^ to demonstrate that magnonic systems can not only achieve tunable ultrastrong coupling, but antiresonant ultrastrong coupling with tunable anisotropy, in a material compatible with ultrafast coherent control. Further, we observed that ∣*g*_2_∣ monotonically increase with *θ*. We found that the anisotropy between *g*_1_ and *g*_2_ depends on magnetic parameters through the spin canting angle, and that the coupling becomes more anisotropic as the canting angle decreases (see Supplementary Note 6, Supplementary Fig. 12). For *θ* = 60^∘^ and 90^∘^, Fig. 4b plots the qFM and qAFM modes (black dashed-dotted lines), the LM and UM (red solid lines), and the co-rotating coupled magnon frequencies (green dashed lines) that are obtained by setting *g*_2_ = 0. The VBSSs are indicated by the shaded areas between the red solid lines and the green dashed lines. We see that for *θ* = 60^∘^, the VBSSs are small relative to the VRSSs (differences between green dashed lines and black dashed-dotted lines), but that the opposite is true when *θ* = 90^∘^. For the UM, the VBSS even becomes dominant when *θ* = 90^∘^, consistent with the increase of ∣*g*_2_∣ relative to ∣*g*_1_∣ with increasing *θ*. Our observation of a dominant VBSS for the UM is unique to the anisotropic Hopfield Hamiltonian and can only be achieved for ∣*g*_2_∣ > ∣*g*_1_∣ (see Methods). Figure 4c plots figures of merit referred to as normalized coupling strengths for 0^∘^ ≤ *θ* ≤ 89^∘^ (the case for 90^∘^ is discussed in Methods). The normalized coupling strength, defined as the ratio of the coupling strength to the frequency where the decoupled qFM and qAFM modes cross (*ω*_0_), determines whether a system is in the USC regime^2,3^. In a system characterized by one coupling strength *g*, the USC regime has been defined as when *g*/*ω*_0_ > 0.1. Thus, we observe that our system can be continuously tuned between no coupling and USC as a function of *θ*, with the maximum experimentally accessible normalized coupling strengths occurring at *θ* = 58^∘^ for 30 T, and are given by ∣*g*_1_∣/*ω*_0_ = 0.26 and ∣*g*_2_∣/*ω*_0_ = 0.39.

Our observation of large counter-rotating interactions is expected to amplify the two-mode vacuum squeezing of the ground state that was discussed in the earliest theoretical study of USC in an isotropic Hopfield Hamiltonian^25^. To demonstrate the capabilities of using YFeO_3_ to realize a magnonic two-mode squeezed vacuum, we evaluate the quantum fluctuations in our system. We first define a generalized magnon annihilation operator $\hat{c}=\alpha \hat{a}+\beta \hat{b}$ and its corresponding quadrature ${\hat{X}}_{\hat{c},\phi }=(\hat{c}{e}^{i\phi }+{\hat{c}}^{\dagger }{e}^{-i\phi })/2$. The standard quantum limit for the fluctuation of ${\hat{X}}_{\hat{c},\phi }$, defined as its variance when evaluated in the decoupled magnon vacuum, is given by 1/4. We numerically investigated the minimum fluctuation in ${\hat{X}}_{\hat{c},\phi }$ evaluated in the ground state of our coupled magnon system and observed a clear suppression below the standard quantum limit.

Figure 4d illustrates this fluctuation suppression below the standard quantum limit of 0 dB, where we numerically searched for the parameters *α*, *β*, and *ϕ* that minimize this fluctuation. An orthogonal operator to $\hat{c}$ also demonstrating squeezing is discussed in the Methods section. We observed that the maximum experimentally achievable squeezing is 5.9 dB, which occurs at 30 T for *θ* = 90^∘^. This strong degree of squeezing is a direct consequence of our large CRTs. Figure 4d demonstrates that the degree of squeezing in our system is easily tunable with applied magnetic field strength and direction, going beyond previous works studying antiferromagnetic magnon squeezing due soley to intrinsic material properties^40^. We note that the squeezing is related to magnetic parameters through *g*_2_ as the counter-rotating interaction is the source of squeezing. Therefore, increasing the anisotropy between *g*_2_ and *g*_1_ (see Supplementary Note 6, Supplementary Fig. 12), leading to stronger counter-rotating interactions, could further amplify the squeezing. We also observed that the quadrature fluctuations approach zero as *θ* approaches 90^∘^ when evaluated at the field strength where the qFM and qAFM modes cross (see Supplementary Fig. 10), suggesting that our system reaches a critical point. We numerically found that complete quadrature fluctuation suppression (i.e., perfect squeezing) is obtained at a critical coupling strength above which the LM becomes gapless, suggesting a magnonic superradiant phase transition.

Our observation of tunable, anisotropic coupling strengths with the CRTs dominating the co-rotating terms demonstrates that magnons in rare-earth orthoferrites serve as an ideal platform for studying many-bodied quantum optical phenomena in extreme regimes of coupling strengths that are inaccessible to traditional photonic systems. In particular, the magnonic ground state describable as a two-mode squeezed vacuum may lead to a pathway for decoherence-free quantum information technology. Perfect magnon squeezing, predicted for a magnonic superradiant phase, will produce a platform of many-body physics to explore the correlation between the quantum phase transitions and the exotic quantum fluctuations.

## Methods

### Sample preparation

Polycrystalline YFeO_3_ was synthesized by conventional solid state reaction using Y_2_O_3_ (99.9%) and Fe_2_O_3_ (99.9%) powders. According to stoichiometric ratios, original reagents were weighted and pulverized with moderate anhydrous ethanol in an agate mortar. Mixtures were sintered at 1300 ^∘^C for 1000 min, then furnace cooled down to room temperature. We continued to grind the presintered sample into powder, pressed it into sheets, reduced the gap between the powder particles, and conducted the second sintering. The sintering temperature and duration were the same as the pre-firing process. The secondary sintered pellets were thoroughly reground, and the polycrystalline powders were pressed into a rod that is 70–80 mm in length and 5–6 mm in diameter by a Hydrostatic Press System under 70 MPa, and then sintered again at 1300 ^∘^C.

Single crystals were grown in the optical floating zone furnace (FZT-10000-H-VI-P-SH, Crystal Systems Corp; Heat source: four 1 kW halogen lamps). During the crystal growth process, we used a YFeO_3_ single crystal as a seed crystal. The molten zone moved upwards at a rate of 3 mm/h with the seed rod (lower shaft) and the feed rod (upper shaft) counter rotating at 30 rpm in airflow by 3 L/min.

We characterized our obtained crystals with a back-reflection Laue camera and X-ray diffraction (XRD). The results show that the sample is a high quality single crystal without impurity phase. We further prepared sheet samples of YFeO_3_ single crystals along the three crystal axis directions for XRD measurement to ensure the accuracy of the crystal directions.

### THz time-domain magnetospectroscopy

We performed THz time-domain magnetospectroscopy by combining 30-T pulsed magnetic fields with THz TDS. The output from an amplified Ti:Sapphire laser (1 kHz, 150 fs, 775 nm, 0.8 mJ, Clark-MXR, Inc., CPA-2001) is divided between THz generation and detection paths. Intense THz is generated using the tilted-pulse-front excitation method^41^ in LiNbO_3_ and is detected using free-space electro-optic (EO) sampling in ZnTe. The incident THz electric field was linearly polarized parallel to the *a*-axis using a wire-grid polarizer. Transmitted THz electric field components parallel (${{\bf{E}}}_{{\rm{THz}}}^{a}$) and perpendicular (${{\bf{E}}}_{{\rm{THz}}}^{b{\rm{-}}c}$) to the incident radiation were identified using a second wire-grid polarizer, then focused onto the detection crystal.

Magnetic fields up to 30 T were generated in the Rice Advanced Magnet with Broadband Optics (RAMBO), a table-top pulsed magnet that combines strong magnetic fields with diverse spectroscopies^27^. A schematic of RAMBO is illustrated in Supplementary Fig. 1 and is discussed in Supplementary Note 1.

Because our magnetic field changes with time, we must rapidly sample the entire THz waveform. We achieve this by implementing single-shot THz detection using a reflective echelon that separates a reflected optical probe pulse into time-delayed beamlets, thereby stretching the optical pulse front^28^. This linearly polarized stretched pulse front overlaps with the entire THz waveform in our detection crystal. The detection crystal is followed by a quarter-wave plate, a Wollaston prism, and imaging optics to separate orthogonal polarization components of the probe, which we use to generate two images of the reflective echelon on a CMOS camera. Supplementary Fig. 2a displays images of the reflective echelon without (top) and with (bottom) THz radiation propagating through the detection crystal. The red dashed box highlights the position of the large-amplitude THz electric field pulse corresponding to *t* = 0 in the time-domain. We describe how we obtain the THz electric field from these images in the Supplementary Note 2.

For quantitative measurements of the THz electric field, we analyze images both in the presence and absence of the THz electric field, yielding a signal and a reference, respectively. Supplementary Fig. 2b displays the signal and reference obtained from the echelon images in Supplementary Fig. 2a, as well as the waveform, obtained by taking the difference of the signal and the reference.

### Magnon mode excitation and characterization

When using linearly polarized incident THz radiation, there are polarization selection rules for the excitation of the two magnon modes: the qFM mode is excited when a component of the THz magnetic field (**H**_THz_) is perpendicular to the weak ferromagnetic moment (**F**), and the qAFM mode is excited when a component of **H**_THz_ is parallel to **F**^22^. These selection rules are derived from solutions to the equations of motion for **S**_1_ and **S**_2_. To extend this analysis to the coupled modes, we numerically investigated the spin dynamics for the LM and UM. Notably, for *θ* = 90^∘^, we found that the dynamics of the qFM mode when **H**_DC_ = 0 and the dynamics of the LM when **H**_DC_ ≠ 0 are almost identical (see Supplementary Fig. 6a, h for a similar comparison). Given that the excitation of the former is forbidden by the selection rule, we also expect the latter to be forbidden.

In general, the transmitted THz electric fields are elliptically polarized^30^ owing to the spin dynamics and the birefriengence of YFeO_3_^42^. In a tilted magnetic field, whether a magnon mode emits predominantly ${{\bf{E}}}_{{\rm{THz}}}^{a}$ or ${{\bf{E}}}_{{\rm{THz}}}^{b{\rm{-}}c}$ polarized light is further complicated by the coupled spin dynamics and the angled cut of the crystal. Therefore, for a given *θ*, whether ${{\bf{E}}}_{{\rm{THz}}}^{a}$ or ${{\bf{E}}}_{{\rm{THz}}}^{b{\rm{-}}c}$ was used to characterize a magnon mode’s frequency as a function of magnetic field depended on which polarization gave a larger signal.

Transmitted THz electric fields at nonzero **H**_DC_ are obtained by taking the difference between (i) the signal measured at nonzero magnetic field and (ii) a reference, where the signal and reference are defined in the previous section. This yields a waveform in the time-domain, the Fourier transform of which reveals the magnon frequency at nonzero **H**_DC_. This method of data analysis was used to characterize all magnon modes discussed in the main text, with the exception of the lower mode (LM) for *θ* = 60^∘^. To study this weak oscillation, the subtracted reference was taken with THz transmitting through the YFeO_3_ crystal at zero magnetic field, as opposed to without THz transmitting through the crystal. This method can be more sensitive because contributions from the large amplitude **E**_THz_ pulse at *t* = 0 are subtracted out. However, because the difference was taken between two THz waveforms that both propagated through the YFeO_3_ crystal, one at zero **H**_DC_ and one at nonzero **H**_DC_, the analyzed THz waveform contains oscillations from magnons measured at both zero and at nonzero **H**_DC_. This limits the ability to characterize the LM at nonzero **H**_DC_ because its frequency will eventually overlap with the upper mode’s (UM’s) zero-field frequency.

Supplementary Fig. 3 shows the complete set of THz magnetospectroscopy measurements for each YFeO_3_ crystal taken at applied field strengths up to 30 T. Each plot indicates the *θ* and the magnon mode being identified. The spectra corresponding to different **H**_DC_ are vertically offset with increasing field strength. The open circles indicate the qFM magnon frequency, the black circles indicate the UM frequency, and the black triangles indicate the LM frequency. The resonance frequencies and their corresponding magnetic fields are plotted in Fig. 3a of the main text. The spectra were zero-padded for smoothing; the frequency resolution of the measurements (1/*T* where *T* is the Fourier-transformed time range) is indicated by error bars in Fig. 3a of the main text.

### Equations of motion and hamiltonian

We start from a microscopic spin model quantitatively describing interactions between the two Fe^3+^ spin sublattices, including symmetric and antisymmetric exchange interactions, single-ion anisotropies, and the Zeeman interaction^22^. In Supplementary Note 3, we derive the equations of motion in terms of **F** = **R**_**1**_ + **R**_**2**_ and **G** = **R**_**1**_ − **R**_**2**_, where **R**_**i**_ are the two spin-sublattice unit vectors. The equations of motion for small displacements of **F** and **G**, given by *δ***F** and *δ***G** are derived as:

2 $$\left[\begin{array}{l}\delta {\dot{F}}_{x}\\ \delta {\dot{F}}_{y}\\ \delta {\dot{G}}_{x}\\ \delta {\dot{G}}_{y}\\ \end{array}\right]=2\gamma \sin {\beta }_{z}\left[\begin{array}{cccc}0&2{A}_{y}&{D}_{yx}&0\\ -2{A}_{x}&0&0&-{D}_{xy}\\ {D}_{xy}&0&0&2{B}_{y}\\ 0&-{D}_{yx}&-2{B}_{x}&0\end{array}\right]\left[\begin{array}{l}\delta {F}_{x}\\ \delta {F}_{y}\\ \delta {G}_{x}\\ \delta {G}_{y}\\ \end{array}\right]$$

which yield two magnonic eigenfrequencies:

3 $$	{\omega }_{\pm }^{2}=\; \frac{{(4\gamma \sin {\beta }_{z})}^{2}}{2}\left({A}_{x}{A}_{y}+{B}_{x}{B}_{y}-\frac{1}{2}{D}_{xy}{D}_{yx}\right.\\ 	 \pm \sqrt{{\left({A}_{x}{A}_{y}+{B}_{x}{B}_{y}-\frac{1}{2}{D}_{xy}{D}_{yx}\right)}^{2}- 4\left({A}_{x}{B}_{y}-\frac{1}{4}{D}_{xy}^{2}\right)\left({A}_{y}{B}_{x}-\frac{1}{4}{D}_{yx}^{2}\right)}\bigg),$$

where *γ* is the gyromagnetic ratio, *β*_*z*_ is the angle between **R**_*i*_ and the *a*-*b* plane, and analytical expressions for *A*_*x*_, *A*_*y*_, *B*_*x*_, *B*_*y*_, *D*_*xy*_, and *D*_*yx*_ in terms of magnetic parameters are provided in the derivation in Supplementary Note 3.

One can show that when **H**_DC_ is parallel to the *c*-axis, *D*_*xy*_ and *D*_*yx*_ exactly vanish. In this geometry, the equations of motion for *δF*_*x*,*y*_ and *δG*_*x*,*y*_ oscillations decouple, becoming the well-known qFM and qAFM modes^22^, respectively. We calculate the generalized, decoupled qFM and qAFM modes in a tilted magnetic field by neglecting *D*_*xy*_ and *D*_*yx*_. To calculate the magnon frequencies plotted in Fig. 3a of the main text, we input magnetic parameters from previous studies of magnons in YFeO_3_^33^.

To fully understand the magnonic interactions, we derive the following quantized Hamiltonian from the microscopic spin model in Supplementary Note 3:

4 $${\mathcal{H}}=\hslash {\omega }_{0a}\left({\hat{a}}^{\dagger }\hat{a}+\frac{1}{2}\right)+\hslash {\omega }_{0b}\left({\hat{b}}^{\dagger }\hat{b}+\frac{1}{2}\right)+i\hslash {g}_{1}\left(\hat{a}{\hat{b}}^{\dagger }-{\hat{a}}^{\dagger }\hat{b}\right)+i\hslash {g}_{2}\left({\hat{a}}^{\dagger }{\hat{b}}^{\dagger }-\hat{a}\hat{b}\right)$$

where $[\hat{a},{\hat{a}}^{\dagger }]=[\hat{b},{\hat{b}}^{\dagger }]=1$, and *ω*_0*a*_ and *ω*_0*b*_ are the decoupled qFM and qAFM magnon frequencies in a general, tilted magnetic field. These are expressed in terms of the parameters in Eq. (2) as:

5 $${\omega }_{0a}=4\gamma \sin {\beta }_{z}\sqrt{{A}_{x}{A}_{y}}$$

6 $${\omega }_{0b}=4\gamma \sin {\beta }_{z}\sqrt{{B}_{x}{B}_{y}}$$

and *g*_1_ and *g*_2_ are the co-rotating and counter-rotating coupling strengths, respectively, expressed as:

7 $${g}_{1}=\gamma \sin {\beta }_{z}\left[{D}_{xy}\left(\frac{{A}_{y}{B}_{x}}{{A}_{x}{B}_{y}}\right)^{1/4}\, -\, {D}_{yx}\left(\frac{{A}_{x}{B}_{y}}{{A}_{y}{B}_{x}}\right)^{1/4}\right]$$

8 $${g}_{2}=\gamma \sin {\beta }_{z}\left[{D}_{xy}\left(\frac{{A}_{y}{B}_{x}}{{A}_{x}{B}_{y}}\right)^{1/4}\, +\, {D}_{yx}\left(\frac{{A}_{x}{B}_{y}}{{A}_{y}{B}_{x}}\right)^{1/4}\right]$$

Importantly, the coupling strengths exactly vanish when the applied magnetic field vanishes or is directed along the *c*-axis. The coupled magnon eigenfrequencies can be derived in terms of *g*_1_, *g*_2_, *ω*_0*a*_, and *ω*_0*b*_ from the equations of motion for the Hamiltonian in Eq. (4), which are provided in Supplementary Note 3. These magnon eigenfrequencies are given by:

9 $${{{\varOmega }}}_{\pm }^{2}=\frac{1}{2}\left[2{g}_{1}^{2}-2{g}_{2}^{2}+{\omega }_{0a}^{2}+{\omega }_{0b}^{2}\pm \sqrt{4{g}_{1}^{2}{({\omega }_{0a}+{\omega }_{0b})}^{2}+{({\omega }_{0a}^{2}-{\omega }_{0b}^{2})}^{2}-4{g}_{2}^{2}{({\omega }_{0a}-{\omega }_{0b})}^{2}}\right]$$

where *Ω*_+_ (*Ω*_−_) is the UM (LM) eigenfrequency, and can be calculated and found to agree exactly with the previously calculated values, thereby confirming our quantized Hamiltonian.

### Symmetry of equations of motion

When **H**_DC_ is applied along the *c*-axis, Eq. (2) becomes block diagonal, and the eigenmodes are given by the well-known qFM and qAFM modes. This block diagonal matrix commutes with:

10 $${{\varSigma }}=\left[\begin{array}{cccc}-1&0&0&0\\ 0&-1&0&0\\ 0&0&1&0\\ 0&0&0&1\end{array}\right]$$

which represents a *π* rotation about the *c*-axis followed by sublattice exchange^32^. Therefore, the qAFM mode is unchanged under the operation of *Σ*, whereas the qFM mode gains a *π* phase shift. However, when **H**_DC_ is tilted in the *b-c* plane, the *π* rotational symmetry of **S**_1_ and **S**_2_ is broken, corresponding to nonzero *β*_*y*_ in Supplementary Fig. 4. Accordingly, *Σ* no longer commutes with the Eq. (2) due to nonzero *D*_*xy*_ and *D*_*yx*_.

As described in the previous section, the generalized qFM and qAFM modes in a tilted magnetic field are defined as independent precessions of *δF*_*x*,*y*_ and of *δG*_*x*,*y*_, respectively, calculated in the absence of *D*_*xy*_ and *D*_*yx*_ in Eq. (2). Thus, they are also eigenstates of *Σ* and their parities (phases) are identical to those for the decoupled qFM and qAFM modes.

### Spin dynamics

We numerically solve the equations of motion for **F** and **G** specified in Eq. (2) for several geometries, which we transform back to the dynamics for the spin sublattice unit vectors **R**_**1**_ and **R**_**2**_. The dynamics for **R**_**i**_ take a simpler form when transformed from Cartesian coordinates (*X*_*i*_, *Y*_*i*_, *Z*_*i*_) to the local, right-handed coordinate system (${S}_{i},{T}_{i},Y^{\prime}$) wherein **R**_**i**_ has components (1, 0, 0) in equilibrium. The transformation is illustrated in Supplementary Fig. 4 and is discussed in Supplementary Note 3. Supplementary Fig. 5 shows the spin dynamics for *θ* = 0^∘^ in the qFM and qAFM modes at applied field strengths of 5 T and 20 T (a–d), and for *θ* = 20^∘^ in the qFM mode, qAFM mode, LM, and UM at an applied field strength of 5 T (e–h). Supplementary Fig. 6 displays the spin dynamics for *θ* = 90^∘^ at applied field strengths of 5 T and 20 T in the qFM mode, qAFM mode, LM, and UM. The spin dynamics in each mode for an applied field strength of 5 T are qualitatively illustrated in plots c, f, i, and l, with the position of each spin on its trajectory indicated in plots a, d, g, and j, respectively.

### Hopfield–Bogoliubov transformation

We perform a Hopfield–Bogoliubov transformation to diagonalize our Hamiltonian, Eq. (4). We introduce coupled magnon annihilation operators $${\hat{B}}_{L}$$$$({\hat{B}}_{U})$$ describing the LM (UM), which are expressed in terms of the generalized qFM (qAFM) operators $$\hat{a}$$ ($$\hat{b}$$) by:

11 $${\hat{B}}_{j}={W}_{j}\hat{a}+{X}_{j}\hat{b}+{Y}_{j}{\hat{a}}^{\dagger }+{Z}_{j}{\hat{b}}^{\dagger }$$

for *j* = *L*, *U*. The coefficients are solutions to an eigenvalue problem discussed in Supplementary Note 4. The Hamiltonian can be rewritten as:

12 $${\mathcal{H}}=\hslash {{{\varOmega }}}_{-}{\hat{B}}_{L}^{\dagger }{\hat{B}}_{L}+\hslash {{{\varOmega }}}_{+}{\hat{B}}_{U}^{\dagger }{\hat{B}}_{U}$$

and the ground state $\left|0\right\rangle$ of our coupled magnon system must satisfy:

13 $${\hat{B}}_{j}\left|0\right\rangle =0$$

### Time-reversed components in lower and upper modes

As shown in Supplementary Fig. 6, the qFM and LM precessions are almost identical. However, the major axes of the qAFM precessions are canted to the *T*_1,2_ axes, while those of the UM are along the ${Y}_{1,2}^{\prime}$ axes. Due to the presence of both co-rotating and the counter-rotating interactions, the coupled magnon dynamics should be a superposition of not only the decoupled qFM and qAFM modes, but also their time-reversals. In the following, we try to understand qualitatively how the time-reversed dynamics are included in the UM.

As seen in Supplementary Fig. 6, the ${Y}_{1,2}^{\prime}$ oscillations (dashed curves) are similar between the qAFM and UM. Thus, we must consider how the small *T*_1,2_ oscillations (solid curves) in the UM are obtained by superposing the qAFM and qFM modes. The UM’s small *T*_1,2_ oscillations (*π*/2 phase-shifted from ${Y}_{1,2}^{\prime}$ oscillations) are already included in the qAFM. They are seen as the small left-shifted *T*_1_ and right-shifted *T*_2_ oscillations in the qAFM. Then, by eliminating the overall large oscillations of *T*_1,2_ (roughly 0 or *π* phase-shifted oscillation from ${Y}_{1,2}^{\prime}$), we get the UM dynamics.

If we eliminate the qAFM’s overall *T*_1,2_ oscillation simply by superposing the dynamics of the qFM, the qFM’s ${Y}_{1,2}^{\prime}$ oscillations (dashed curves) are also added. They are in-phase with each other. However, the UM’s ${Y}_{1,2}^{\prime}$ oscillations are out-of-phase with each other. So, the simple superposition of the qAFM and qFM cannot reproduce the UM dynamics.

The solution is the superposition not only with the qFM but also with the time-reversed qFM. The superposition of the qFM and its time-reversal (1→2→3→4 and 3→2→1→4) has only the *T*_1,2_ oscillations (${Y}_{1,2}^{\prime}$ oscillations are eliminated). Then, by superposing both the qFM and its time-reversal, the qAFM is transformed to the UM.

We quantitatively check the weight of the time-reversed qFM in the UM by evaluating the coefficients from the Hopfield–Bogoliubov transformation Eq. (11). Supplementary Fig. 7a shows the weights of the qFM (∣*W*_*U*_∣^2^) and qAFM (∣*X*_*U*_∣^2^) modes, and Supplementary Fig. 7b shows those of the time-reversed qFM (∣*Y*_*U*_∣^2^) and time-reversed qAFM (∣*Z*_*U*_∣^2^) modes, all in the UM as functions of the applied field strength for *θ* = 90^∘^. The qFM mode (∣*W*_*U*_∣^2^) and its time-reversal (∣*Y*_*U*_∣^2^) have the same weight, consistent with the above discussion.

Supplementary Fig. 7c, d show the same weights in the LM. While the spin dynamics of the qFM and LM are quite similar as discussed above, we observe that the LM also contains large time-reversed components. The ${Y}_{1,2}^{\prime}$ amplitudes in the LM are in fact slightly larger (about 4%) than those in the qFM. The time-reversed qFM and qAFM are required for reproducing this difference.

### Squeezing

To demonstrate that $\left|0\right\rangle$ satisfying ${\hat{B}}_{L}\left|0\right\rangle ={\hat{B}}_{U}\left|0\right\rangle =0$ is an intrinsically quantum vacuum squeezed state, we first introduce two orthogonal, generalized annihilation operators:

14 $$\hat{c}=\alpha \hat{a}+\beta \hat{b}$$

15 $$\hat{d}={\beta }^{* }\hat{a}-\alpha \hat{b}$$

where $\alpha \in {\mathbb{R}}$, $\beta \in {\mathbb{C}}$ and they satisfy *α*^2^ + ∣*β*∣^2^ = 1. With respect to these generalized annihilation operators, we define the following quadratures:

16 $${\hat{X}}_{\hat{c},\phi }=(\hat{c}{e}^{i\phi }+{\hat{c}}^{\dagger }{e}^{-i\phi })/2$$

17 $${\hat{X}}_{\hat{d},\phi }=(\hat{d}{e}^{i\phi }+{\hat{d}}^{\dagger }{e}^{-i\phi })/2$$

The standard quantum limit for both of these operators is given by 1/4.

The variances of ${\hat{X}}_{\hat{c},\phi }$ and ${\hat{X}}_{\hat{d},\phi }$ can be easily evaluated in $\left|0\right\rangle$ by inverting the Hopfield–Bogoliubov transformation and rewriting the quadratures in terms of ${\hat{B}}_{j}$. Expressions for these variances are provided in Supplementary Note 5. Using these expressions, we minimized the quadrature variances by numerically searching for the optimal *α*, *β*, and *ϕ*. Supplementary Fig. 8 shows the fluctuation suppression for *θ* = 20^∘^, 40^∘^, 60^∘^, and 90^∘^. Note that we did not observe squeezing for the case when *θ* = 0^∘^.

### Squeezing and phase transition

To evaluate the contribution of *g*_2_ to the squeezing, we numerically calculated the minimum quadrature variance $\langle 0| {({{\Delta }}{\hat{X}}_{\hat{c},\phi })}^{2}| 0\rangle$ while artificially changing ∣*g*_2_∣ and keeping the other parameters as obtained at *θ* = 90^∘^ and 30 T. In Supplementary Fig. 9a, the minimum $\langle 0| {({{\Delta }}{\hat{X}}_{\hat{c},\phi })}^{2}| 0\rangle$ is plotted as a function of ∣*g*_2_∣. The minimum quadrature variance is increased to 0.25 (0 dB) at ∣*g*_2_∣ = 0. By increasing ∣*g*_2_∣, one can find that the minimum quadrature variance drops to zero at ∣*g*_2_∣ = 2*π* × 0.763 THz.

This condition corresponds to the superradiant phase transition when we transform our anisotropic Hopfield Hamiltonian, Eq. (4), into the anisotropic Dicke Hamiltonian, given by:

18 $${\mathcal{H}}\to \hslash {\omega }_{0a}\left({\hat{a}}^{\dagger }\hat{a}+\frac{1}{2}\right)+\hslash {\omega }_{0b}\left({\hat{S}}_{z}+\frac{N}{2}\right)+\frac{i\hslash {g}_{1}}{\sqrt{N}}(\hat{a}{\hat{S}}_{+}-{\hat{a}}^{\dagger }{\hat{S}}_{-})+\frac{i\hslash {g}_{2}}{\sqrt{N}}\Big({\hat{a}}^{\dagger }{\hat{S}}_{+}-\hat{a}{\hat{S}}_{-}\Big)$$

Here, ${\hat{S}}_{x,y,z}$ are the spin-$\frac{N}{2}$ operator, and ${\hat{S}}_{\pm }\equiv {\hat{S}}_{x}\pm i{\hat{S}}_{y}$ are the raising and lowering operators. The phase transition is obtained in this Hamiltonian when the LM’s eigenfrequency becomes zero, indicating an instability of the normal phase. This condition is derived from Eq. (9) as:

19 $$1+\frac{{({g}_{{1}}^{2}-{g}_{{2}}^{2})}^{2}}{{\omega }_{{0a}}^{2}{\omega }_{{0b}}^{2}}-\frac{2({g}_{{1}}^{2}+{g}_{{2}}^{2})}{{\omega }_{0a}{\omega }_{0b}}=0$$

In the isotropic case *g*_1_ = *g*_2_ = *g*, this is reduced to the well-known condition 4*g*^2^ = *ω*_0*a*_*ω*_0*b*_ of the superradiant phase transition in the isotropic Dicke Hamiltonian.

The drop condition ∣*g*_2_∣ = 2*π* × 0.763 THz of the minimum quadrature variance in Supplementary Fig. 9a satisfies Eq. (19). In this way, the minimum quadrature variance becomes zero at the superradiant phase transition.

### Comparison of VBSS and VRSS

We define the VBSS and VRSS for the UM as:

20 $${\rm{VBSS}}={{{\varOmega }}}_{+}({g}_{1}\,\ne\,0,{g}_{2}=0)-{{{\varOmega }}}_{+}({g}_{1}\,\ne\,0,{g}_{2}\,\ne\,0)$$

21 $${\rm{VRSS}}={{{\varOmega }}}_{+}({g}_{1}\,\ne\, 0,{g}_{2}=0)-\max ({\omega }_{0a},{\omega }_{0b})$$

Here, we assume that $\max ({\omega }_{0a},{\omega }_{0b})={\omega }_{0b}$, but similar results can be derived for $\max ({\omega }_{0a},{\omega }_{0b})={\omega }_{0a}$.

The condition for VBSS > VRSS is derived from Eq. (9) as:

22 $$2{\omega }_{0a}{\omega }_{0b}({g}_{1}^{2}+{g}_{2}^{2})\,<\,({g}_{2}^{2}-{g}_{1}^{2})\left[({g}_{2}^{2}-{g}_{1}^{2})+2{\omega }_{0b}^{2}\right]$$

We can immediately identify that this cannot be satisfied in the isotropic case where ${g}_{1}^{2}={g}_{2}^{2}$. The condition for the normal phase, from Eq. (19), is given by:

23 $${g}_{1}^{2}-{g}_{2}^{2}\,<\,{\omega }_{0a}{\omega }_{0b}-\sqrt{4{g}_{2}^{2}{\omega }_{0a}{\omega }_{0b}}$$

Under the assumption that ∣*g*_1_∣ > ∣*g*_2_∣, one can only satisfy Eq. (22) if:

24 $${g}_{1}^{2}-{g}_{2}^{2}\,> \,{\omega }_{0b}({\omega }_{0a}+{\omega }_{0b})+\sqrt{4{g}_{2}^{2}{\omega }_{0a}{\omega }_{0b}+{\omega }_{0b}^{2}{({\omega }_{0a}+{\omega }_{0b})}^{2}}$$

Thus, for ∣*g*_1_∣ > ∣*g*_2_∣, one cannot achieve VBSS > VRSS for the UM in the normal phase. However, for ∣*g*_2_∣ > ∣*g*_1_∣, the condition for VBSS > VRSS can be derived as:

25 $${g}_{2}^{2}\,> \,{g}_{1}^{2}-{\omega }_{0b}({\omega }_{0b}-{\omega }_{0a})+\sqrt{{\omega }_{0b}^{2}{({\omega }_{0b}-{\omega }_{0a})}^{2}+4{\omega }_{0a}{\omega }_{0b}{g}_{1}^{2}}\quad (> {g}_{1}^{2})$$

which can be satisfied in the normal phase.

### Discontinuity for *θ* = 90^∘^

To calculate normalized coupling strengths presented in Fig. 4c of the main text, we require the magnetic field *H*_cross_ at which the generalized qFM and qAFM mode frequencies cross. Supplementary Fig. 10a plots calculated values of *H*_cross_ for all 0^∘^ ≤ *θ* ≤ 90^∘^.

We observe that at an applied field strength of 1284 T for *θ* = 90^∘^, a magnetically driven phase transition occurs, where S_1_ and S_2_ become perfectly aligned along the *b*-axis. We also find that the generalized qFM and qAFM magnon frequencies, as well as the coupling strengths *g*_1_ and *g*_2_, are unstable at this point and change discontinuously, leading to a discontinuity in the normalized coupling strengths, illustrated in Supplementary Fig. 10b.

## Supplementary information

Supplementary Information

## Data Availability

Source data are provided with this paper. All other data that support the plots within this paper and other findings of this study are available from the corresponding authors upon reasonable request.

## Source data

Source Data

## Code availability

The codes that support the plots within this paper and other findings of this study are available from the corresponding authors upon reasonable request.
