# Supplementary material for: Ultrastrong magnon–magnon coupling dominated by antiresonant interactions
Source: Nat Commun. 2021 May 25;12:3115. doi: 10.1038/s41467-021-23159-z (PMC8149649; doi:10.1038/s41467-021-23159-z)
Supplement: Supplementary file 1 — Supplementary Information [file 41467_2021_23159_MOESM1_ESM.pdf]

Supplementary Information for
Ultrastrong Magnon-Magnon Coupling Dominated by Antiresonant Interactions

Takuma Makihara,[1] Kenji Hayashida,[2,3] G. Timothy Noe II,[2] Xinwei Li,[2] Nicolas Marquez Peraca,[1] Xiaoxuan Ma,[4] Zuanming Jin,[5] Wei Ren,[4] Guohong Ma,[4] Ikufumi Katayama,[6] Jun Takeda,[6] Hiroyuki Nojiri,[7] Dmitry Turchinovich,[8] Shixun Cao,[4,*] Motoaki Bamba,[9,10,*] Junichiro Kono[1,2,11,*]

Supplementary Note 1: Rice Advanced Magnet with Broadband Optics (RAMBO)

RAMBO is schematically illustrated in Supplementary Fig. 1. Magnetic fields are generated using a 30 T pulsed mini-coil with a 12 mm inner diameter. The magnet is housed in a custom-built liquid nitrogen cryostat that serves to prevent Joule heating from changing the resistance of the coil between magnetic field pulses and to lower the resistance of the coil, thereby increasing the peak magnetic field strength. Coaxial electrodes connect the mini-coil to a 5.6 mF capacitor bank capable of being charged up to over $1,800$ V, as is required for 30 T magnetic field pulses. Single crystals of $YFeO_3$ are held in the center of the mini-coil by being mounted on the narrow end of a tapered sapphire pipe (minimum inner diameter 6 mm, minimum outer diameter 8 mm). The sapphire pipe is attached to the copper coldfinger of a liquid helium cryostat by an indium ring. The coldfinger is covered by a radiation shield to prevent heating from the cryostat walls. With these measures in place, the lowest sample temperature achievable is roughly 12 K, although the measurements presented in this work were done without liquid helium and were therefore done at room temperature. The liquid helium cryostat and liquid nitrogen cryostat are combined by a bellows joint, enabling fine adjustments of the sample's position in the bore of the magnet.

Optical access to the sample is provided through the tapered sapphire pipe and windows on the liquid helium and liquid nitrogen cryostats. The tapering of the sapphire pipe increases the optical access to the sample. The magnetic field profile is determined by measuring the current delivered to the mini-coil using a Rogowski Coil (Powertek, CWT 60 Mini Rogowski Coil). The relationship between the current delivered to the mini-coil and the magnetic field profile was calibrated using a pickup coil placed at center of the bore of the magnet. To determine the temporal relationship between laser pulses from our amplified Ti:Sapphire and the magnetic field profile, a photodiode is placed adjacent to the THz generation crystal to measure scattered light. By using single-shot THz detection, we are able to measure transmitted THz electric fields at three different

magnetic field strengths for a single magnetic field pulse, as illustrated in Fig. 1c of the main text.

Supplementary Note 2: Single-shot Detection

The transmitted THz waveform is obtained from the image of the reflective echelon following our procedure in Reference[1]. For each column of pixels imaged on the CMOS camera, the total intensity of one polarization component is calculated by vertically integrating over one half of the column, and the total intensity of the other polarization component is calculated by vertically integrating over the other half of the column. The difference of these two intensities is labeled ΔI. Although the optical probe pulse is magnified before the echelon to ensure uniform intensity over the image, we normalize both polarization components' intensities by the total intensity of the column (I_o) to normalize for nonuniform probe intensity over the echelon. The ratio $\Delta I/I_o$, is proportional to the THz electric field strength. This process is repeated for all columns in order to find the THz electric field strength at each horizontal pixel location on the camera, as indicated in Supplementary Fig. 2. To calibrate horizontal pixel position with time, the optical probe pulse is delayed by a known time, causing the THz electric field position on the echelon to move a known number of pixels, yielding a pixel-time calibration.

Supplementary Note 3: Derivation of Equations of Motion, Hamiltonian, and Magnon Eigenfrequencies

We start from the treatment given by Herrmann[2] by considering interactions between the two Fe^{3+} spin sublattices. The free energy of the system, normalized by the magnitude M_0 of the two spin sublattices $\mathbf{S_1}$ and $\mathbf{S_2}$, was first derived by Herrmann for the Γ_4 phase with an applied magnetic field along the c-axis. The normalized free energy accounting for a tilted magnetic field in the $b-c$

plane is given by:

$$V = E\mathbf{R_1}\cdot\mathbf{R_2} - D(X_1Z_2 - X_2Z_1) - A_{xx}(X_1^2 + X_2^2) - A_{zz}(Z_1^2 + Z_2^2)$$
$$- H_y(Y_1 + Y_2) - H_z(Z_1 + Z_2) \tag{1}$$

where $\mathbf{R_i} = \mathbf{S_i}/M_0 = (X_i, Y_i, Z_i)$ for $(i = 1, 2)$ are the spin sublattice unit vectors, E is the isotropic exchange constant, D is the antisymmetric exchange constant, A_{xx} and A_{zz} are anisotropy constants, and H_y (H_z) is the component of the applied magnetic field ($\mathbf{H}_{\mathrm{DC}}$) along the $b-$ ($c-$) axis. Note that we assume the anisotropy constant A_{xz} to be zero for simplicity, as in References[3–5]. In equilibrium, in the Γ_4 phase, $\mathbf{R_1}$ and $\mathbf{R_2}$ are ordered antiferromagnetically along the a-axis, with a slight canting towards the c-axis. In terms of the ferromagnetic vector $\mathbf{F} = \mathbf{R_1} + \mathbf{R_2}$ and the antiferromagnetic vector $\mathbf{G} = \mathbf{R_1} - \mathbf{R_2}$, we have that $\mathbf{F}$ points along the c-axis, and $\mathbf{G}$ points along the a-axis.

The equations of motion for $\mathbf{R_i}$ are given by:

$$-\frac{1}{\gamma}\dot{\mathbf{R}}_\mathbf{i} = \mathbf{R_i} \times \nabla_i V \tag{2}$$

where γ is the gyromagetic ratio and $\nabla_i V$ is the gradient of V with respect to (X_i, Y_i, Z_i). The equations of motion for $\mathbf{R_i}$ take a simpler form when transformed from Cartesian coordinates (X_i, Y_i, Z_i) to the local, right-handed coordinate system (S_i, T_i, Y_i') wherein $\mathbf{R_i}$ has components $(1, 0, 0)$ in equilibrium. The transformation, as illustrated in Supplementary Fig. 4, is given by:

$$\begin{cases} X_1 = S_1\cos\beta_z\cos\beta_y + T_1\sin\beta_z\cos\beta_y - Y_1'\sin\beta_y \\ Y_1 = S_1\cos\beta_z\sin\beta_y + T_1\sin\beta_z\sin\beta_y + Y_1'\cos\beta_y \\ Z_1 = S_1\sin\beta_z - T_1\cos\beta_z \end{cases} \tag{3}$$

$$\begin{cases} X_2 = -S_2\text{cos}\beta_z\text{cos}\beta_y + T_2\text{sin}\beta_z\text{cos}\beta_y + Y_2'\text{sin}\beta_y \\ Y_2 = S_2\text{cos}\beta_z\text{sin}\beta_y - T_2\text{sin}\beta_z\text{sin}\beta_y + Y_2'\text{cos}\beta_y \\ Z_2 = S_2\text{sin}\beta_z + T_2\text{cos}\beta_z \end{cases} \tag{4}$$

The equilibrium positions are found by solving the equations of motion for $\dot{\mathbf{R}}_\mathbf{i} = 0$.

Expanding the equations of motion about their equilibrium position and retaining only linear terms in small fluctuations of T_i and Y_i', represented by δT_i and $\delta Y_i'$, results in the equations of motion:

$$\frac{1}{\gamma}\begin{bmatrix} \delta\dot{T}_1 \\ \delta\dot{Y}_1' \\ \delta\dot{T}_2 \\ \delta\dot{Y}_2' \end{bmatrix} = \begin{bmatrix} -\frac{\partial^2 V}{\partial Y_1' \partial T_1} & \langle\frac{\partial V}{\partial S_1}\rangle - \frac{\partial^2 V}{\partial Y_1'^2} & -\frac{\partial^2 V}{\partial Y_1' \partial T_2} & -\frac{\partial^2 V}{\partial Y_1' \partial Y_2'} \\ \frac{\partial^2 V}{\partial T_1^2} - \langle\frac{\partial V}{\partial S_1}\rangle & \frac{\partial^2 V}{\partial T_1 \partial Y_1'} & \frac{\partial^2 V}{\partial T_1 \partial T_2} & \frac{\partial^2 V}{\partial T_1 \partial Y_2'} \\ -\frac{\partial^2 V}{\partial Y_2' \partial T_1} & -\frac{\partial^2 V}{\partial Y_2' \partial Y_1'} & -\frac{\partial^2 V}{\partial Y_2' \partial T_2} & \langle\frac{\partial V}{\partial S_2}\rangle - \frac{\partial^2 V}{\partial Y_2'^2} \\ \frac{\partial^2 V}{\partial T_2 \partial T_1} & \frac{\partial^2 V}{\partial T_2 \partial Y_1'} & \frac{\partial^2 V}{\partial T_2^2} - \langle\frac{\partial V}{\partial S_2}\rangle & \frac{\partial^2 V}{\partial T_2 \partial Y_2'} \end{bmatrix} \begin{bmatrix} \delta T_1 \\ \delta Y_1' \\ \delta T_2 \\ \delta Y_2' \end{bmatrix} \tag{5}$$

where $\langle ... \rangle$ represents an evaluation of the derivative at the equilibrium position. For notational simplicity, we rewrite Supplementary Eq. 5 as:

$$\frac{1}{\gamma}\begin{bmatrix} \delta\dot{T}_1 \\ \delta\dot{Y}_1' \\ \delta\dot{T}_2 \\ \delta\dot{Y}_2' \end{bmatrix} = \begin{bmatrix} -e & -a & -f & b \\ -c & e & -d & g \\ -g & b & -h & -a \\ -d & f & -c & h \end{bmatrix} \begin{bmatrix} \delta T_1 \\ \delta Y_1' \\ \delta T_2 \\ \delta Y_2' \end{bmatrix} \tag{6}$$

which defines the constants a, b, c, d, e, f, g, and h. These are given by:

$$
\begin{aligned}
a &= E(\cos^2\beta_z\cos 2\beta_y - \sin^2\beta_z) + D\sin 2\beta_z\cos\beta_y + 2A_{xx}\cos^2\beta_z\cos^2\beta_y \\
&\quad + 2A_{zz}\sin^2\beta_z + H_y\cos\beta_z\sin\beta_y + H_z\sin\beta_z + \left(-2A_{xx}\sin^2\beta_y\right) \\
b &= -E\cos 2\beta_y \\
c &= -E(\cos^2\beta_z\cos 2\beta_y - \sin^2\beta_z) - D\sin 2\beta_z\cos\beta_y \\
&\quad - 2A_{xx}\cos 2\beta_z\cos^2\beta_y + 2A_{zz}\cos 2\beta_z - H_y\cos\beta_z\sin\beta_y - H_z\sin\beta_z \\
d &= E(\cos^2\beta_z - \sin^2\beta_z\cos 2\beta_y) + D\sin 2\beta_z\cos\beta_y \\
e &= A_{xx}\sin\beta_z\sin 2\beta_y \\
f &= D\cos\beta_z\sin\beta_y - E\sin\beta_z\sin 2\beta_y \\
g &= -D\cos\beta_z\sin\beta_y + E\sin\beta_z\sin 2\beta_y = -f \\
h &= -A_{xx}\sin\beta_z\sin 2\beta_y = -e
\end{aligned}
\tag{7}
$$

Given that δT_i and $\delta Y_i'$ are canonical variables, the equations of motion given in Supplementary Eq. 5 are recovered from Hamilton's equations:

$$
\begin{cases}
\delta\dot{T}_i = \frac{1}{\hbar}\frac{\partial\mathcal{H}}{\partial(\delta Y_i')} \\
\delta\dot{Y}_i' = -\frac{1}{\hbar}\frac{\partial\mathcal{H}}{\partial(\delta T_i)}
\end{cases}
\tag{8}
$$

for the following Hamiltonian:

$$
\begin{aligned}
\frac{\mathcal{H}}{\hbar\gamma} &= -\frac{a}{2}((\delta Y_1')^2 + (\delta Y_2')^2) + \frac{c}{2}((\delta T_1)^2 + (\delta T_2)^2) + b\delta Y_1'\delta Y_2' + d\delta T_1\delta T_2 \\
&\quad - e\delta T_1\delta Y_1' - f\delta Y_1'\delta T_2 - g\delta T_1\delta Y_2' - h\delta T_2\delta Y_2'
\end{aligned}
\tag{9}
$$

The equations of motion and their physical interpretation are more illuminating when rewrit-

ten in terms of **F** and **G**. The transformation is given by:

$$\begin{cases} \delta F_x = (\delta T_1 + \delta T_2)\sin\beta_z\cos\beta_y - (\delta Y_1' - \delta Y_2')\sin\beta_y \\ \delta F_y = (\delta T_1 - \delta T_2)\sin\beta_z\sin\beta_y + (\delta Y_1' + \delta Y_2')\cos\beta_y \\ \delta F_z = -(\delta T_1 - \delta T_2)\cos\beta_z \end{cases} \tag{10}$$

$$\begin{cases} \delta G_x = (\delta T_1 - \delta T_2)\sin\beta_z\cos\beta_y - (\delta Y_1' + \delta Y_2')\sin\beta_y \\ \delta G_y = (\delta T_1 + \delta T_2)\sin\beta_z\sin\beta_y + (\delta Y_1' - \delta Y_2')\cos\beta_y \\ \delta G_z = -(\delta T_1 + \delta T_2)\cos\beta_z \end{cases} \tag{11}$$

and the transformed Hamiltonian is given by:

$$\frac{\mathcal{H}}{\hbar\gamma} = A_x\delta F_x^2 + A_y\delta F_y^2 + B_x\delta G_x^2 + B_y\delta G_y^2 + D_{xy}\delta F_x\delta G_y + D_{yx}\delta F_y\delta G_x \tag{12}$$

where the coefficients are functions of the spin sublattice equilibrium positions and derivatives of the free energy, and are given by:

$$\begin{aligned} A_x &= \frac{-1}{4}(b+a)\sin^2\beta_y + \frac{1}{4\sin^2\beta_z}(d+c)\cos^2\beta_y + \frac{1}{4\sin\beta_z}(e+f-g-h)\sin\beta_y\cos\beta_y \\ A_y &= \frac{1}{4}(b-a)\cos^2\beta_y - \frac{1}{4\sin^2\beta_z}(d-c)\sin^2\beta_y + \frac{1}{4\sin\beta_z}(-e+f-g+h)\sin\beta_y\cos\beta_y \\ B_x &= \frac{1}{4}(b-a)\sin^2\beta_y - \frac{1}{4\sin^2\beta_z}(d-c)\cos^2\beta_y + \frac{1}{4\sin\beta_z}(e-f+g-h)\sin\beta_y\cos\beta_y \\ B_y &= \frac{-1}{4}(b+a)\cos^2\beta_y + \frac{1}{4\sin^2\beta_z}(d+c)\sin^2\beta_y + \frac{1}{4\sin\beta_z}(-e-f+g+h)\sin\beta_y\cos\beta_y \\ D_{xy} &= (\frac{a}{2} + \frac{c}{2\sin^2\beta_z} + \frac{b}{2} + \frac{d}{2\sin^2\beta_z})\sin\beta_y\cos\beta_y - \frac{1}{4\sin\beta_z}(e+f-g-h)\cos2\beta_y \\ D_{yx} &= (\frac{a}{2} + \frac{c}{2\sin^2\beta_z} - \frac{b}{2} - \frac{d}{2\sin^2\beta_z})\sin\beta_y\cos\beta_y - \frac{1}{4\sin\beta_z}(e-f+g-h)\cos2\beta_y \end{aligned} \tag{13}$$

Since we get

$$[\delta F_x, \delta F_y] = [\delta G_x, \delta G_y] = i2\sin\beta_z \tag{14}$$

from Supplementary Eq. 10, Supplementary Eq. 11, $[\delta T_i, \delta Y'_j] = i\delta_{i,j}$, and $[\delta T_i, \delta T_j] = [\delta Y'_i, \delta Y'_j] = 0$, we consider δF_x and $\delta F_y/2\sin\beta_z$ (δG_x and $\delta G_y/2\sin\beta_z$) to be canonically conjugate variables. Hamilton's equations for δF_x and $\delta F_y/2\mathrm{sin}\beta_z$ are given by:

$$\begin{cases} \delta\dot{F}_x = \frac{1}{\hbar}\frac{\partial\mathcal{H}}{\partial(\delta F_y/2\mathrm{sin}\beta_z)} = 2\gamma\mathrm{sin}\beta_z(2A_y\delta F_y + D_{yx}\delta G_x) \\ \frac{\delta\dot{F}_y}{2\mathrm{sin}\beta_z} = \frac{-1}{\hbar}\frac{\partial\mathcal{H}}{\partial(\delta F_x)} = -\gamma(2A_x\delta F_x + D_{xy}\delta G_y) \end{cases} \tag{15}$$

and similarly for δG_x and $\delta G_y/2\mathrm{sin}\beta_z$:

$$\begin{cases} \delta\dot{G}_x = \frac{1}{\hbar}\frac{\partial\mathcal{H}}{\partial(\delta G_y/2\mathrm{sin}\beta_z)} = 2\gamma\mathrm{sin}\beta_z(2B_y\delta G_y + D_{xy}\delta F_x) \\ \frac{\delta\dot{G}_y}{2\mathrm{sin}\beta_z} = \frac{-1}{\hbar}\frac{\partial\mathcal{H}}{\partial(\delta G_x)} = -\gamma(2B_x\delta G_x + D_{yx}\delta F_y) \end{cases} \tag{16}$$

which can be written more compactly as:

$$\begin{bmatrix} \delta\dot{F}_x \\ \delta\dot{F}_y \\ \delta\dot{G}_x \\ \delta\dot{G}_y \end{bmatrix} = 2\gamma\mathrm{sin}\beta_z \begin{bmatrix} 0 & 2A_y & D_{yx} & 0 \\ -2A_x & 0 & 0 & -D_{xy} \\ D_{xy} & 0 & 0 & 2B_y \\ 0 & -D_{yx} & -2B_x & 0 \end{bmatrix} \begin{bmatrix} \delta F_x \\ \delta F_y \\ \delta G_x \\ \delta G_y \end{bmatrix} \tag{17}$$

These equations of motion yield two magnonic eigenfrequencies:

$$\omega_\pm^2 = \frac{(4\gamma\mathrm{sin}\beta_z)^2}{2}\Bigg(A_xA_y + B_xB_y - \frac{1}{2}D_{xy}D_{yx} \pm \sqrt{(A_xA_y + B_xB_y - \frac{1}{2}D_{xy}D_{yx})^2 - 4(A_xB_y - \frac{1}{4}D_{xy}^2)(A_yB_x - \frac{1}{4}D_{yx}^2)}\Bigg) \tag{18}$$

as discussed in Methods.

To fully understand magnonic interactions, we proceed to quantize our Hamiltonian. Here, we introduce magnonic creation and annihilation operators for each magnon mode: $\hat{a}$, $\hat{a}^\dagger$ for the generalized qFM mode and $\hat{b}$, $\hat{b}^\dagger$ for the generalized qAFM mode. We may rewrite fluctuations in

$\mathbf{F}$ in terms of these new operators:

$$\begin{cases} \delta F_x = \sin\beta_z \big(\frac{A_y}{A_x \sin^2\beta_z}\big)^{1/4} (\hat{a}^\dagger + \hat{a}) \\ \delta F_y = i \big(\frac{A_x \sin^2\beta_z}{A_y}\big)^{1/4} (\hat{a}^\dagger - \hat{a}) \end{cases} \tag{19}$$

Similarly, we may rewrite fluctuations in $\mathbf{G}$ in terms of $\hat{b}$ and $\hat{b}^\dagger$:

$$\begin{cases} \delta G_x = \sin\beta_z \big(\frac{B_y}{B_x \sin^2\beta_z}\big)^{1/4} (\hat{b}^\dagger + \hat{b}) \\ \delta G_y = i \big(\frac{B_x \sin^2\beta_z}{B_y}\big)^{1/4} (\hat{b}^\dagger - \hat{b}) \end{cases} \tag{20}$$

Supplementary Eq. 14 is certainly satisfied from $[\hat{a}, \hat{a}^\dagger] = [\hat{b}, \hat{b}^\dagger] = 1$. We can rewrite our Hamiltonian, Supplementary Eq. 12, in terms of the magnonic creation and annihilation operators:

$$\mathcal{H} = \hbar\omega_{0a}(\hat{a}^\dagger \hat{a} + \frac{1}{2}) + \hbar\omega_{0b}(\hat{b}^\dagger \hat{b} + \frac{1}{2}) + i\hbar g_1(\hat{a}\hat{b}^\dagger - \hat{a}^\dagger \hat{b}) + i\hbar g_2(\hat{a}^\dagger \hat{b}^\dagger - \hat{a}\hat{b}) \tag{21}$$

where ω_{0a} and ω_{0b} are generalized qFM and qAFM magnon frequencies given by:

$$\omega_{0a} = 4\gamma \sin\beta_z \sqrt{A_x A_y} \tag{22}$$

$$\omega_{0b} = 4\gamma \sin\beta_z \sqrt{B_x B_y} \tag{23}$$

and g_1 and g_2 are the co-rotating and counter-rotating coupling strengths, respectively, given by:

$$g_1 = \gamma \sin\beta_z \left[D_{xy} \big(\frac{A_y B_x}{A_x B_y}\big)^{1/4} - D_{yx} \big(\frac{A_x B_y}{A_y B_x}\big)^{1/4} \right] \tag{24}$$

$$g_2 = \gamma \sin\beta_z \left[D_{xy} \big(\frac{A_y B_x}{A_x B_y}\big)^{1/4} + D_{yx} \big(\frac{A_x B_y}{A_y B_x}\big)^{1/4} \right] \tag{25}$$

We confirm our quantized Hamiltonian by calculating its predicted magnon frequencies and comparing it to our previously derived magnon frequencies. The magnon frequencies are obtained

from the equations of motion for the creation and annihilation operators:

$$\begin{cases} \frac{d\hat{a}(t)}{dt} = \frac{1}{i\hbar}[\hat{a}(t), \mathcal{H}] \\ \frac{d\hat{b}(t)}{dt} = \frac{1}{i\hbar}[\hat{b}(t), \mathcal{H}] \\ \frac{d\hat{a}^\dagger(t)}{dt} = \left(\frac{d\hat{a}(t)}{dt}\right)^\dagger \\ \frac{d\hat{b}^\dagger(t)}{dt} = \left(\frac{d\hat{b}(t)}{dt}\right)^\dagger \end{cases} \tag{26}$$

which yields the following equations of motion:

$$\frac{d}{dt}\begin{bmatrix} \hat{a}(t) \\ \hat{b}(t) \\ \hat{a}^\dagger(t) \\ \hat{b}^\dagger(t) \end{bmatrix} = \begin{bmatrix} -i\omega_{0a} & -g_1 & 0 & g_2 \\ g_1 & -i\omega_{0b} & g_2 & 0 \\ 0 & g_2 & i\omega_{0a} & -g_1 \\ g_2 & 0 & g_1 & i\omega_{0b} \end{bmatrix} \begin{bmatrix} \hat{a}(t) \\ \hat{b}(t) \\ \hat{a}^\dagger(t) \\ \hat{b}^\dagger(t) \end{bmatrix} \tag{27}$$

Expressions for the two positive magnonic eigenfrequencies are given by:

$$\Omega_\pm^2 = \frac{1}{2}[2g_1^2 - 2g_2^2 + \omega_{0a}^2 + \omega_{0b}^2 \pm \sqrt{4g_1^2(\omega_{0a} + \omega_{0b})^2 + (\omega_{0a}^2 - \omega_{0b}^2)^2 - 4g_2^2(\omega_{0a} - \omega_{0b})^2}] \tag{28}$$

Supplementary Note 4: Hopfield-Bogoliubov Transformation

Here, we diagonalize our Hamiltonian using a Hopfield-Bogoliubov transformation. We introduce coupled magnon annihilation operators $\hat{B}_L$ ($\hat{B}_U$) describing the LM (UM), which are expressed in terms of the generalized qFM (qAFM) operators $\hat{a}$ ($\hat{b}$) by:

$$\hat{B}_j = W_j\hat{a} + X_j\hat{b} + Y_j\hat{a}^\dagger + Z_j\hat{b}^\dagger \tag{29}$$

for $j = L, U$. The coefficients are constrained by the relation:

$$|W_j|^2 + |X_j|^2 - |Y_j|^2 - |Z_j|^2 = 1 \tag{30}$$

and are determined by the following eigenvalue problem:

$$\begin{bmatrix} \omega_{0a} & ig_1 & 0 & ig_2 \\ -ig_1 & \omega_{0b} & ig_2 & 0 \\ 0 & ig_2 & -\omega_{0a} & ig_1 \\ ig_2 & 0 & -ig_1 & -\omega_{0b} \end{bmatrix} \begin{bmatrix} W_j \\ X_j \\ Y_j \\ Z_j \end{bmatrix} = \Omega_\pm \begin{bmatrix} W_j \\ X_j \\ Y_j \\ Z_j \end{bmatrix} \tag{31}$$

where Ω_+ (Ω_-) corresponds to the UM (LM) eigenfrequency and is provided in Supplementary Eq. 28.

Supplementary Note 5: Evaluating Squeezing

The variances of $\hat{X}_{\hat{c},\phi}$ and $\hat{X}_{\hat{d},\phi}$, defined in Methods, can be easily evaluated in the coupled magnon ground state $|0\rangle$ by inverting the Hopfield-Bogoliubov transformation given in Supplementary Eq. 29 and rewriting the quadratures in terms of $\hat{B}_j$. The fluctuation in $\hat{X}_{\hat{c},\phi}$ in the coupled magnon ground state is given by:

$$\langle 0|(\Delta\hat{X}_{\hat{c},\phi})^2|0\rangle = \sum_{j=L,U} \frac{|\alpha W_j + \beta^* X_j|^2 + |\alpha Y_j + \beta Z_j|^2}{4} - |V|\cos(\varphi - 2\phi) \tag{32}$$

$$V \equiv \sum_{j=L,U} \frac{(\alpha W_j + \beta^* X_j)(\alpha Y_j + \beta Z_j)^*}{2} = |V|e^{i\varphi} \tag{33}$$

and a similar expression can be derived for $\langle 0|(\Delta\hat{X}_{\hat{d},\phi})^2|0\rangle$.

Supplementary Note 6: Origin of $g_{1,2}$ and relation to magnetic parameters

Here, we discuss the origin of the magnon-magnon coupling, $g_{1,2}$, and their relation to magnetic parameters, E, D, A_{xx}, A_{zz}.

First, we clarify that the origin of $g_{1,2}$ is the tilted magnetic field. As discussed in the main text, when $\mathbf{H}_{\mathrm{DC}}$ is zero or applied along the c-axis, the two spin sublattices maintain π rotational

symmetry about the c-axis. Given fixed and opposite parities of the qFM and qAFM modes under this symmetry, their hybridization is prevented in this geometry. When β_y is nonzero, their hybridization is no longer forbidden, where β_y defines how far the spins are pulled from the $a-c$ plane (Supplementary Fig. 4). This is also clear from the analytical definitions of the coupling strengths (Supplementary Eqs. 24, 25), where it follows from Supplementary Eq. 7 and Supplementary Eq. 13 that $g_{1,2}$ exactly vanishes when β_y is zero. Therefore, nonzero β_y is the origin of our magnon-magnon coupling, and we achieve this condition by applying a strong magnetic field with a nonzero component along the b-axis.

Second, we discuss the relation between magnetic parameters and the anisotropy of g_1 and g_2. Large magnitudes of g_2 are crucial for the exotic quantum vacuum phenomena predicted in the ultrastrong coupling regime, and thus there is great motivation for understanding how the strength of these counter-rotating interactions depend on magnetic parameters. Supplementary Fig. 12 shows numerical calculations for normalized coupling strengths $|g_{1,2}|/\omega_0$ vs. θ, where ω_0 is the frequency at which the generalized qFM and qAFM modes cross (also defined in Fig. 3c of the main text). In each plot, we calculate $|g_{1,2}|/\omega_0$ vs. θ as we tune one magnetic parameter. Specifically, in each plot the dotted lines correspond to scaling the chosen magnetic parameter by 2, the dashed lines correspond to scaling the chosen magnetic parameter by 0.5, and the solid lines correspond to the true value of the chosen magnetic parameter (i.e. scaled by 1). In Supplementary Fig. 12a, 12b, 12c, and 12d, we tune E, A_{xx}, D, and A_{zz}, respectively.

From Supplementary Fig. 12, we see that g_2/g_1 becomes larger as E, A_{xx} increase and as D, A_{zz} decrease i.e. the coupling becomes more anisotropic. Similarly, we see that g_2/g_1 becomes smaller as E, A_{xx} decrease and as D, A_{zz} increase i.e. the coupling becomes more isotropic. The relationship between the anisotropicity and the magnetic parameters becomes more illuminating when we recall that increasing E, A_{xx} and decreasing D, A_{zz} reduces the spin canting angle (β_z

in Supplementary Fig. 4), whereas decreasing E, A_{xx} and increasing D, A_{zz} increases the spin canting angle. The relationship between the spin canting angle and magnetic parameters can be analytically derived by solving for the steady states of Supplementary Eq. 2. When $\mathbf{H}_{\text{DC}}$ is applied along the b-axis, one can show that the expression is given by:

$$\tan\beta_z = \left| c_0 \sqrt{\frac{1-Y^2}{1+c_0^2 Y^2}} \right| \tag{34}$$

$$Y = \frac{H_y}{Dc_0 + 2(E + A_{xx})} \tag{35}$$

$$c_0 = \frac{-(E + A_{xx} - A_{zz}) + \sqrt{(E + A_{xx} - A_{zz})^2 + D^2}}{D} \tag{36}$$

where H_y is the b-component of $\mathbf{H}_{\text{DC}}$. Therefore, the coupling becomes more isotropic as the spins become more canted, and the coupling becomes more anisotropic as the spins become more antiparallel. Finally, we highlight that the origin of our ground state squeezing is the counter-rotating terms. Therefore, as the coupling becomes more anisotropic due to stronger counter-rotating interactions the degree of squeezing will also be amplified.

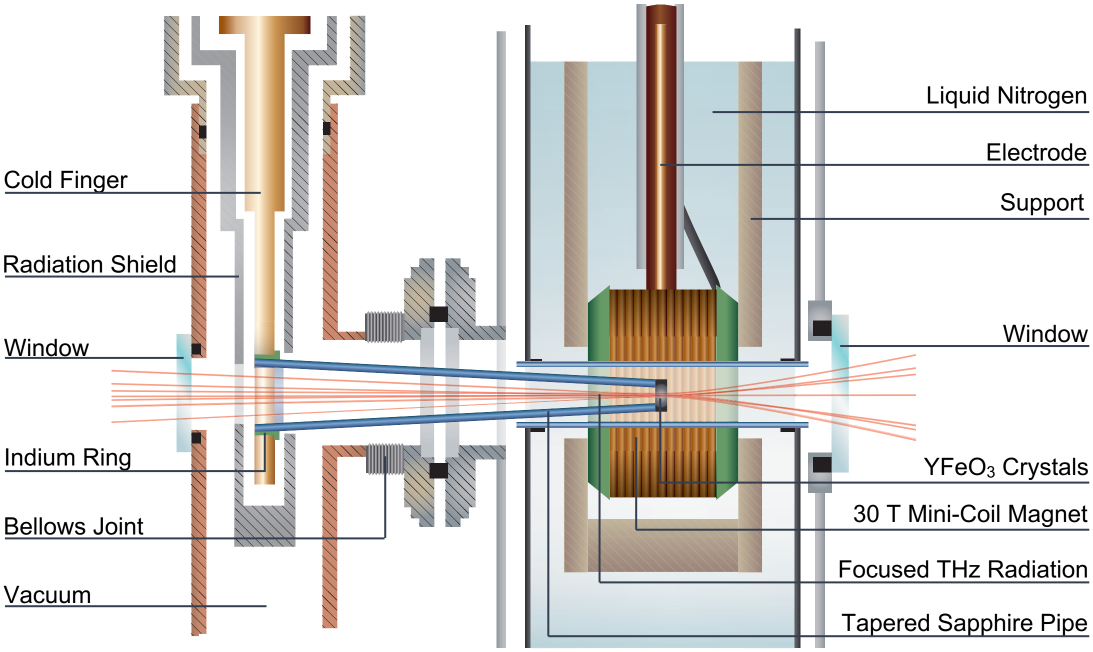

Supplementary Fig. 1. Schematic of RAMBO. Samples are held on the narrow end of a tapered sapphire pipe in the center of a 30 T mini-coil magnet. The magnet is housed in a custom-built liquid nitrogen cryostat and is connected to a capacitor bank by co-axial electrodes. The tapered sapphire pipe is held by an indium ring on the copper cold finger of a liquid helium cryostat. The cold finger is covered by a radiation shield to limit heating from the cryostat walls. The liquid helium and liquid nitrogen cryostats are connected by a bellows joint that enables fine adjustment of the sample position in the magnet.

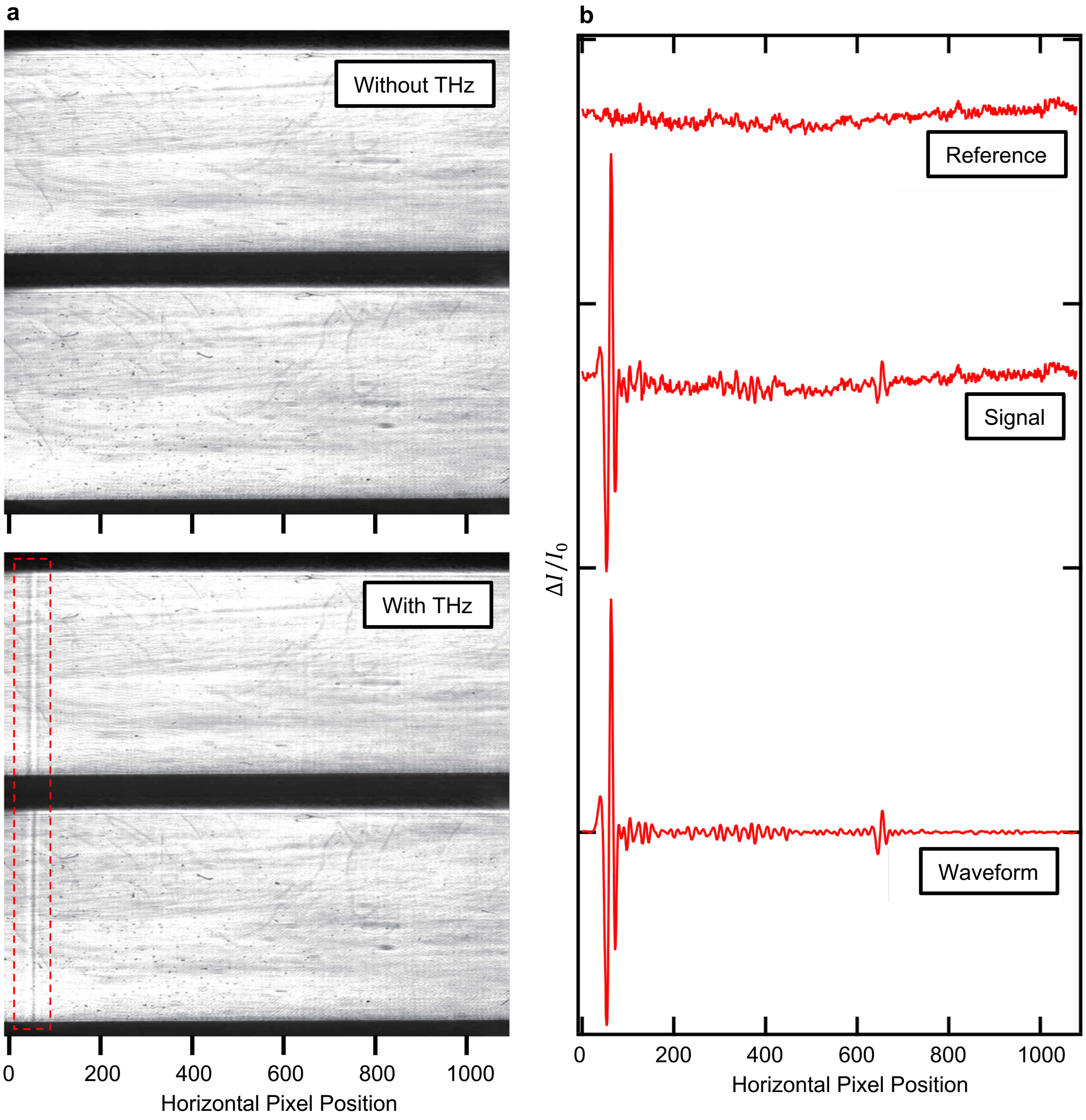

Supplementary Fig. 2. THz waveform extraction from reflective echelon images. a, Images from the CMOS camera of the reflective echelon in the absence (top) and presence (bottom) of THz radiation. The top and bottom halves of each camera image correspond to orthogonal polarization components of the stretched probe pulse front used in single-shot detection. The red dashed box highlights the position of the large amplitude E_{THz} pulse centered at $t = 0$. **b,** The *reference* obtained by analyzing the top CMOS camera image, the *signal* obtained by analyzing the bottom CMOS camera image, and the *waveform* obtained from taking their difference, where the CMOS camera images are those shown in **a**.

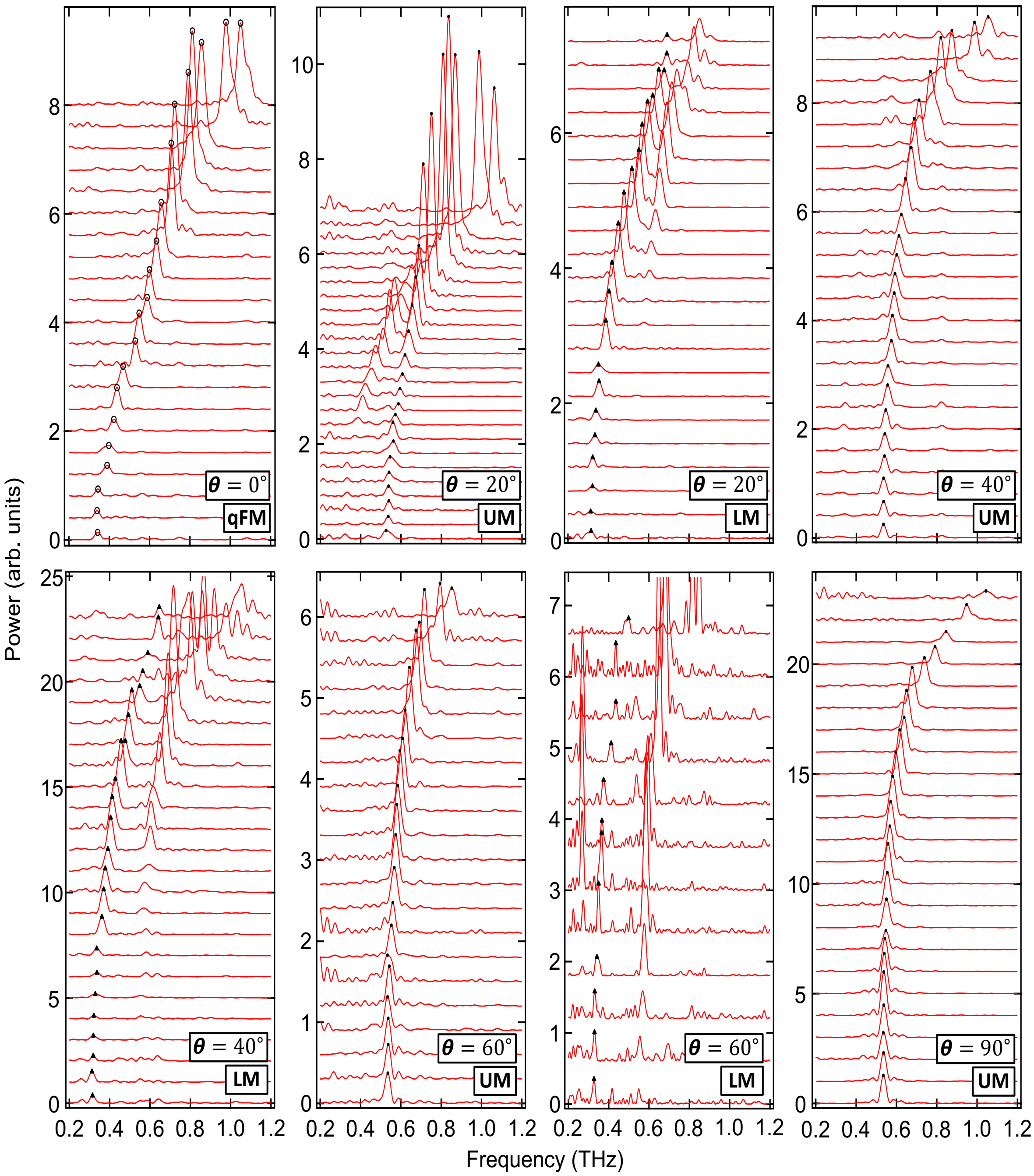

Supplementary Fig. 3. Complete set of THz magnetospectroscopy measurements of $YFeO_3$. Each plot shows the angle θ between $\mathbf{H}_{\text{DC}}$ and the c-axis in the $b-c$ plane, as well as the magnon mode being studied. Spectra measured at different field strengths are vertically offset for clarity with increasing field strength, where the magnon frequency and field strength are shown in Fig. 3a of the main text. Open circles indicate the qFM mode, black circles indicate the UM, and the black triangles indicate the LM.

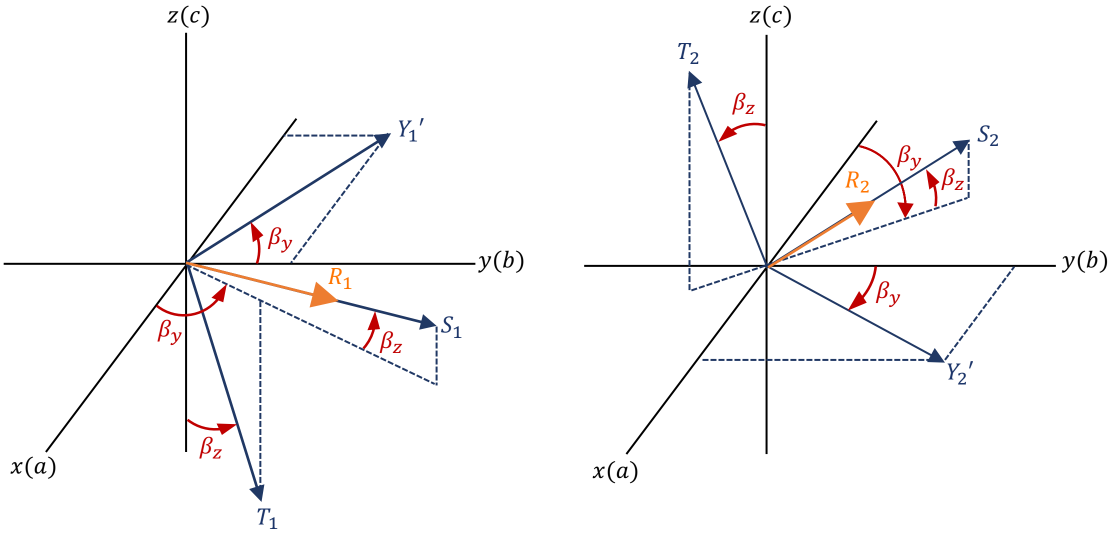

Supplementary Fig. 4. Equilibrium position of R_1 and R_2 transformed to local, right-handed coordinates. The right-handed coordinates (S_i, T_i, Y_i') in which $\mathbf{R}_i$ (for $i = 1, 2$) has coordinates (1, 0, 0) in equilibrium, for the case of a general applied magnetic field direction in the $b - c$ plane.

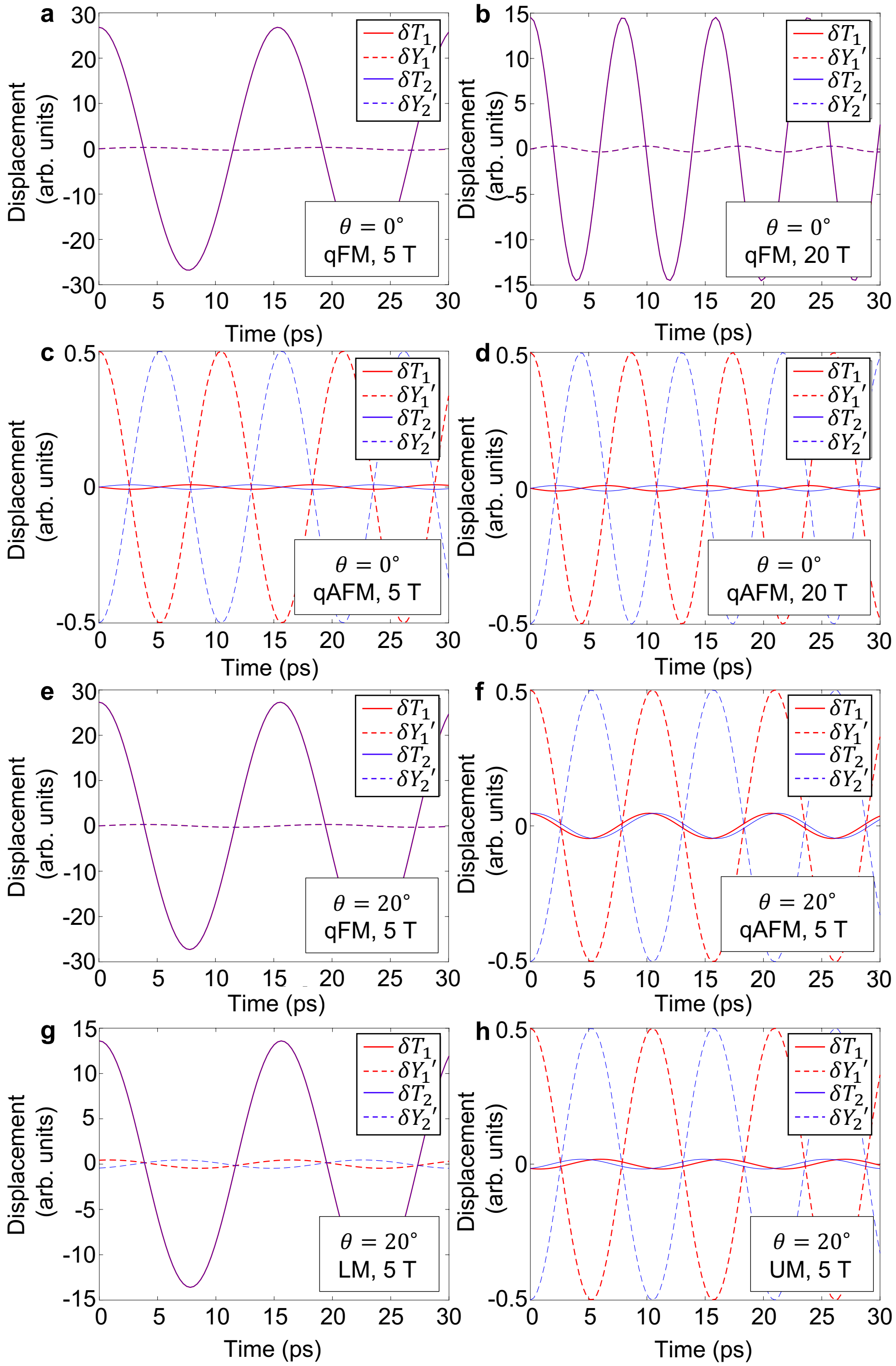

Supplementary Fig. 5. Spin dynamics for $\theta = 0°$ and $\theta = 20°$. a-d, Dynamics of $\delta T_{1,2}$ and $\delta Y'_{1,2}$ for $\theta = 0°$ in the qFM and qAFM modes at applied field strengths of 5 T and 20 T. **e-h,** Dynamics of $\delta T_{1,2}$ and $\delta Y'_{1,2}$ for $\theta = 20°$ in the qFM mode, qAFM mode, LM, and UM at an applied field strength of 5 T.

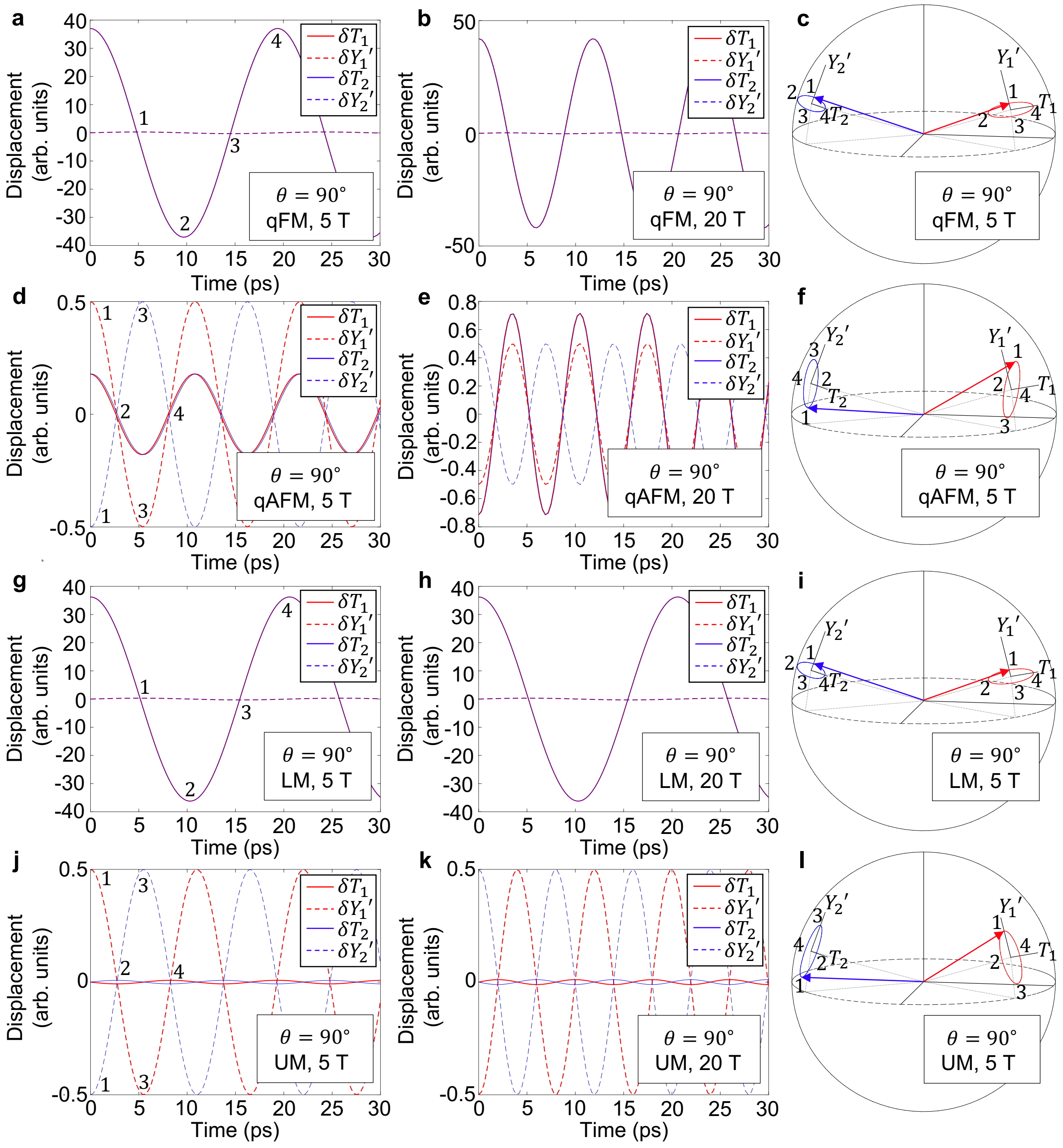

Supplementary Fig. 6. Spin dynamics for $\theta = 90°$. Dynamics of $\delta T_{1,2}$ and $\delta Y'_{1,2}$ for $\theta = 90°$ in the qFM mode (**a-c**), the qAFM mode (**d-f**), the LM (**g-i**), and the UM (**j-l**). Plots **c**, **f**, **i**, **l** qualitatively illustrate the spin dynamics for the modes in **a**, **d**, **g**, and **j**, respectively, with the illustrated spin position at a given time indicated in the corresponding plot.

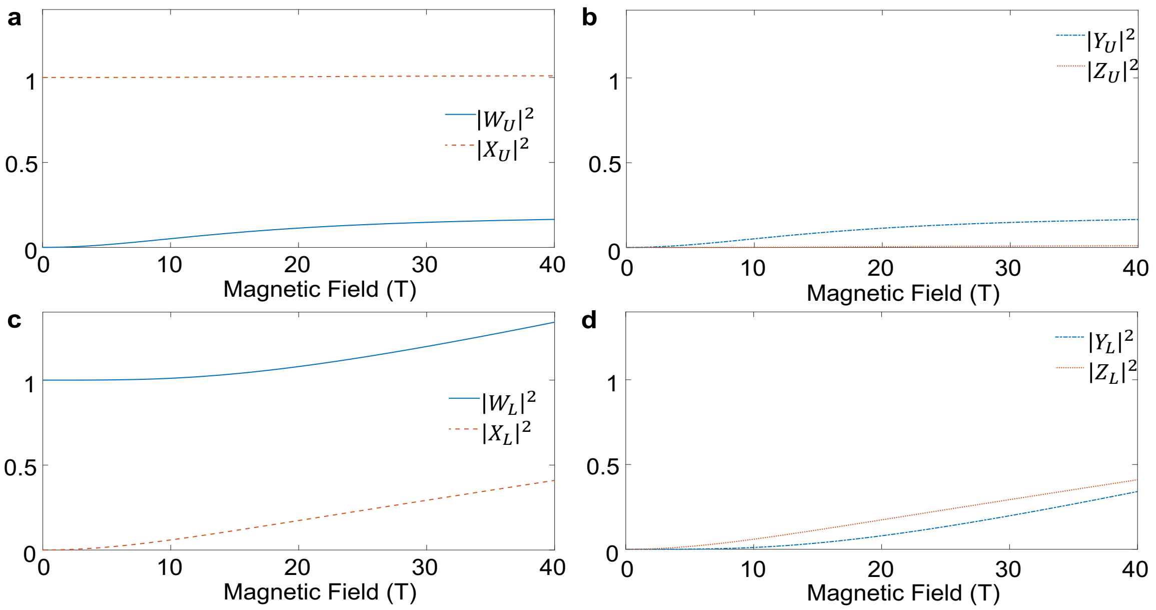

Supplementary Fig. 7. Weights of qFM, qAFM, time-reversed qFM, and time-reversed qAFM modes in the UM and LM. a, The weights of the qFM mode ($|W_U|^2$) and qAFM mode ($|X_U|^2$) in the UM. **b,** The weights of the time-reversed qFM mode ($|Y_U|^2$) and time-reversed qAFM mode ($|Z_U|^2$) in the UM. **c,** The weights of the qFM mode ($|W_L|^2$) and qAFM mode ($|X_L|^2$) in the LM. **d,** The weights of the time-reversed qFM mode ($|Y_L|^2$) and time-reversed qAFM mode ($|Z_L|^2$) in the LM. The probability amplitudes W_j, X_j, Y_j, Z_j are calculated from the Hopfield-Bogoliubov transformation discussed in the Supplementary Information.

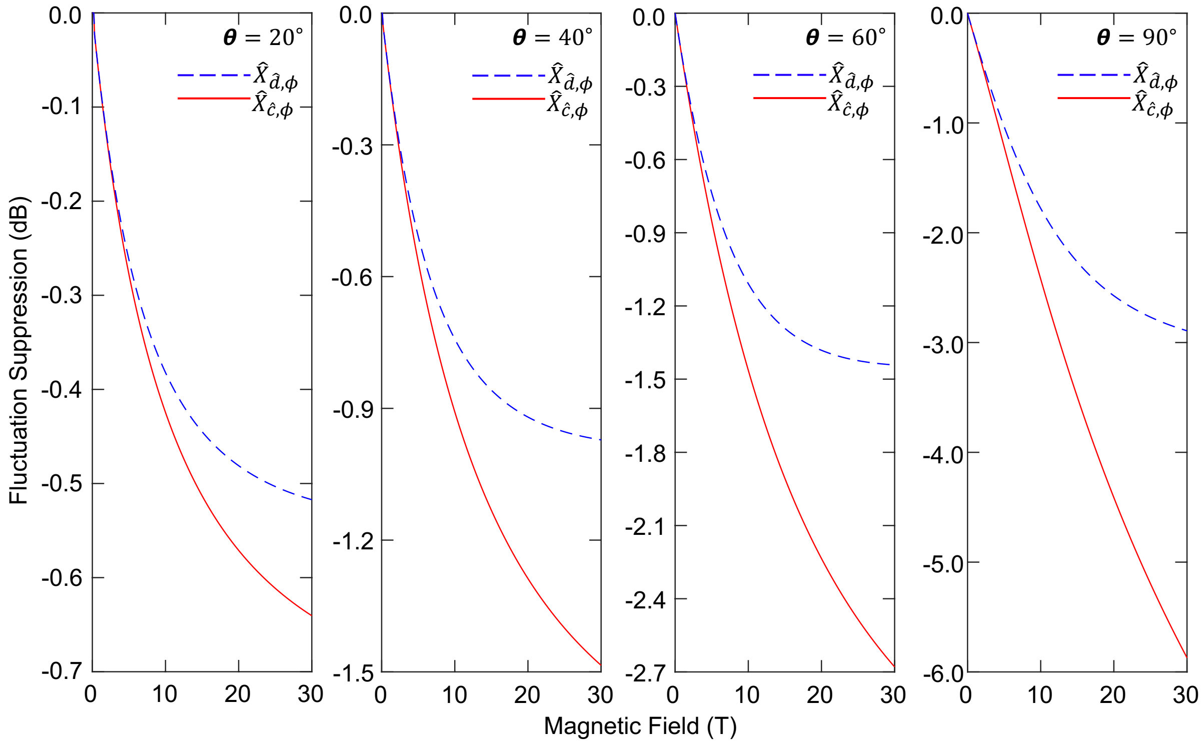

Supplementary Fig. 8. Calculation of Two-Mode Squeezing. Fluctuation suppression in $\langle 0|(\Delta\hat{X}_{\hat{c},\phi})^2|0\rangle$ (red solid lines) and $\langle 0|(\Delta\hat{X}_{\hat{d},\phi})^2|0\rangle$ (blue dashed lines) for $\theta = 20°$, $40°$, $60°$, and $90°$.

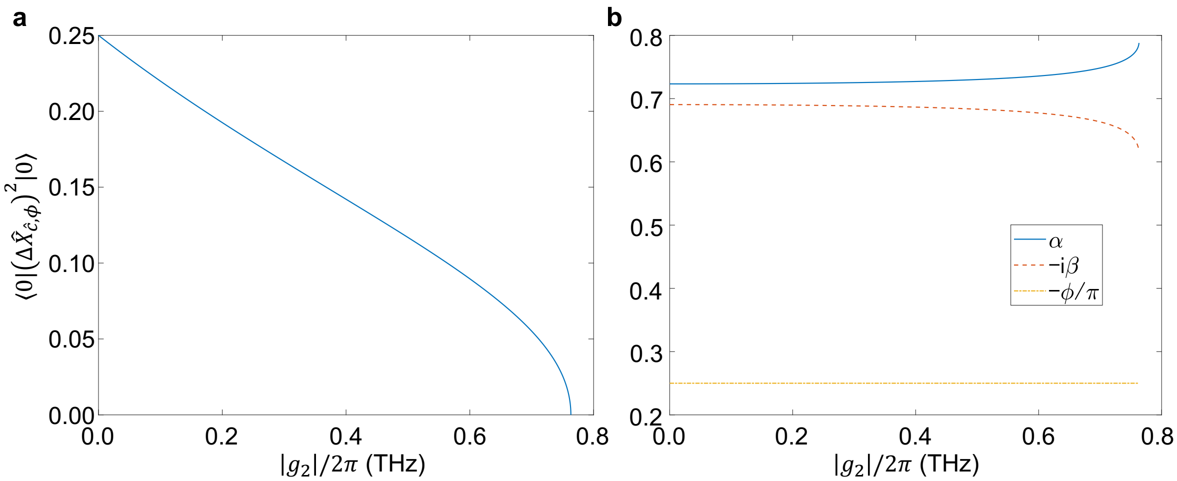

Supplementary Fig. 9. Evidence for perfect squeezing at quantum superradiant phase transition. a, The minimum quadrature variance $\langle 0|(\Delta\hat{X}_{\hat{c},\phi})^2|0\rangle$ calculated as a function of $|g_2|$. **b,** The optimum parameters that give the minimum variance, plotted as functions of $|g_2|$. The minimum quadrature variance drops to zero at $|g_2| = 2\pi \times 0.763$ THz, which corresponds to the "quantum" superradiant phase transition. The other parameters are fixed as those for 30 T and $\theta = 90°$; $\omega_{0a} = 2\pi \times 0.722$ THz, $\omega_{0b} = 2\pi \times 1.201$ THz, and $g_1 = 2\pi \times 0.168$ THz.

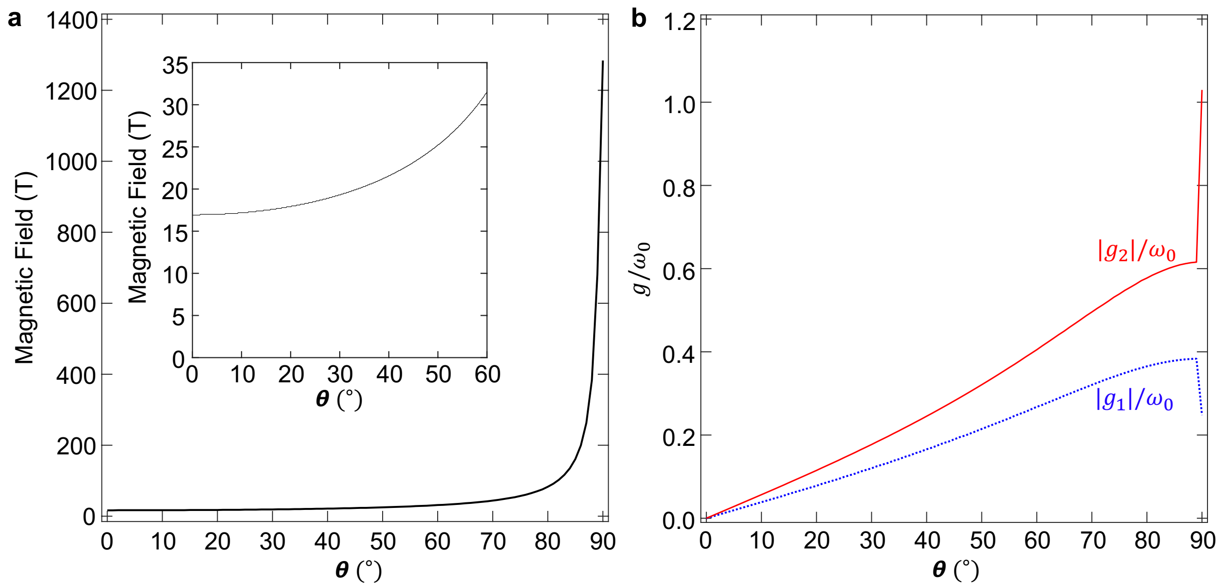

Supplementary Fig. 10. Magnetic field for normalized coupling and discontinuity for $\theta = 90^\circ$. a, Magnetic field at which the generalized qFM and qAFM magnon frequencies cross. **b,** Normalized coupling strengths for $0^\circ \leq \theta \leq 90^\circ$.

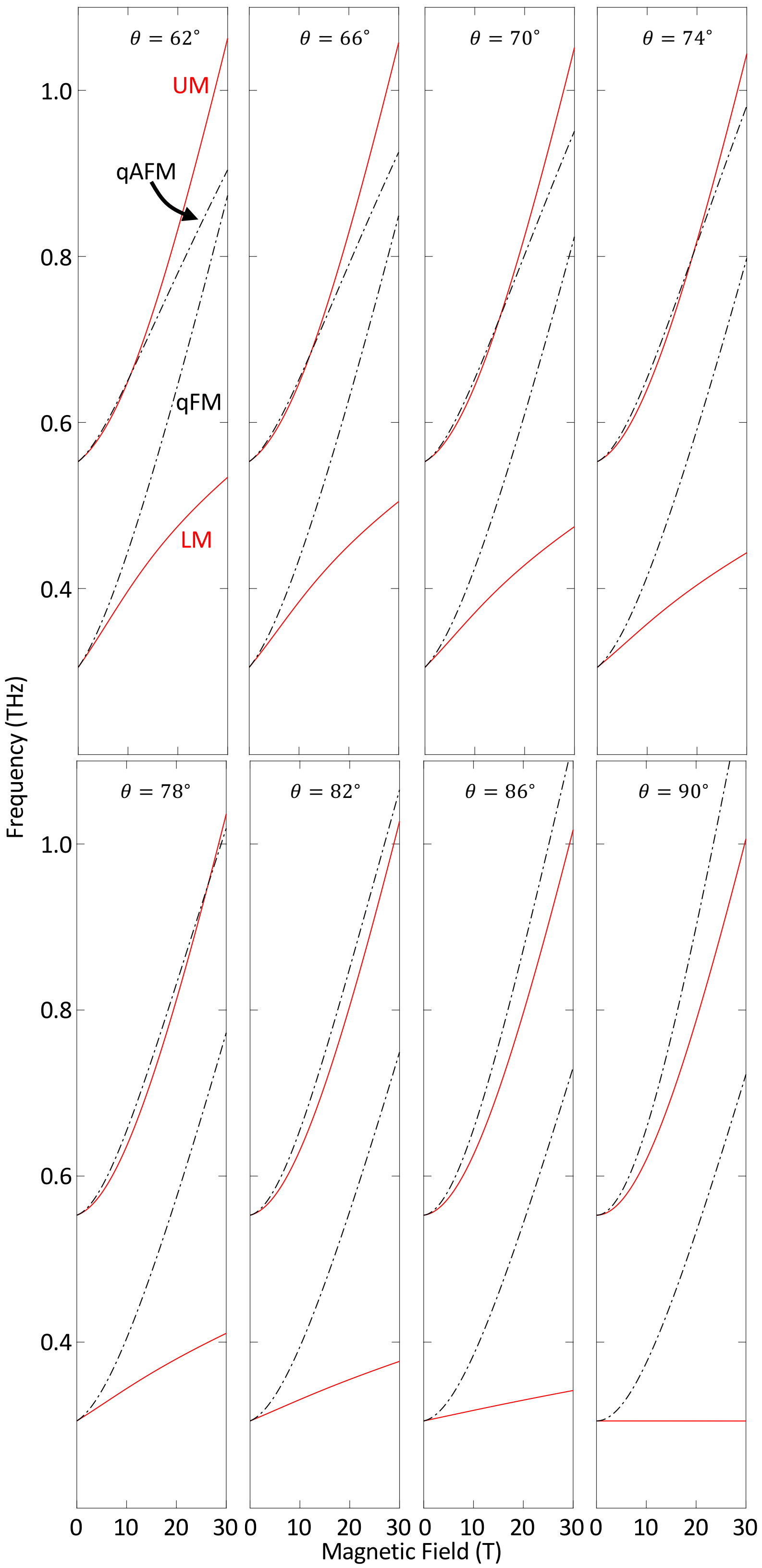

Supplementary Fig. 11. Transition from $\theta = 60°$ **to** $90°$**.** Additional plots showing the transition as the VBSS becomes dominant and pushes the UM below the qAFM mode.

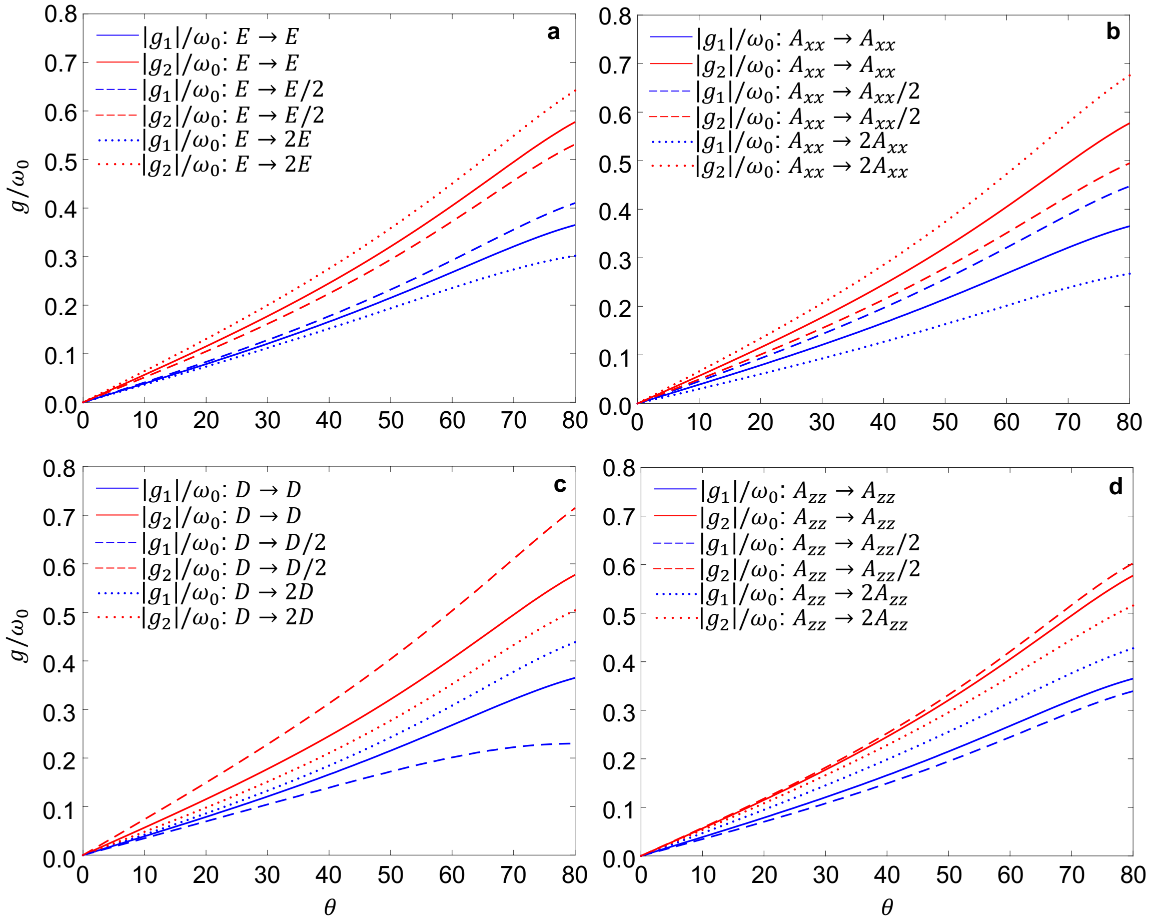

Supplementary Fig. 12. Normalized coupling strengths vs. θ with different magnetic parameters. Plots of normalized coupling strengths $|g_{1,2}|/\omega_0$ vs. θ where in each plot, one magnetic parameter is tuned. The parameter E, A_{xx}, D, A_{zz} is tuned in plot **a**, **b**, **c**, **d**, respectively. In each plot, we recalculate the normalized coupling strengths for when the magnetic parameter is doubled (dotted lines, $E \to 2E$ in **a**) and halved (dashed lines, $E \to E/2$ in **a**), as well as for the true value of the magnetic parameter (solid lines, $E \to E$ in **a**). In each plot, blue lines show $|g_1|/\omega_0$ and red lines show $|g_2|/\omega_0$.

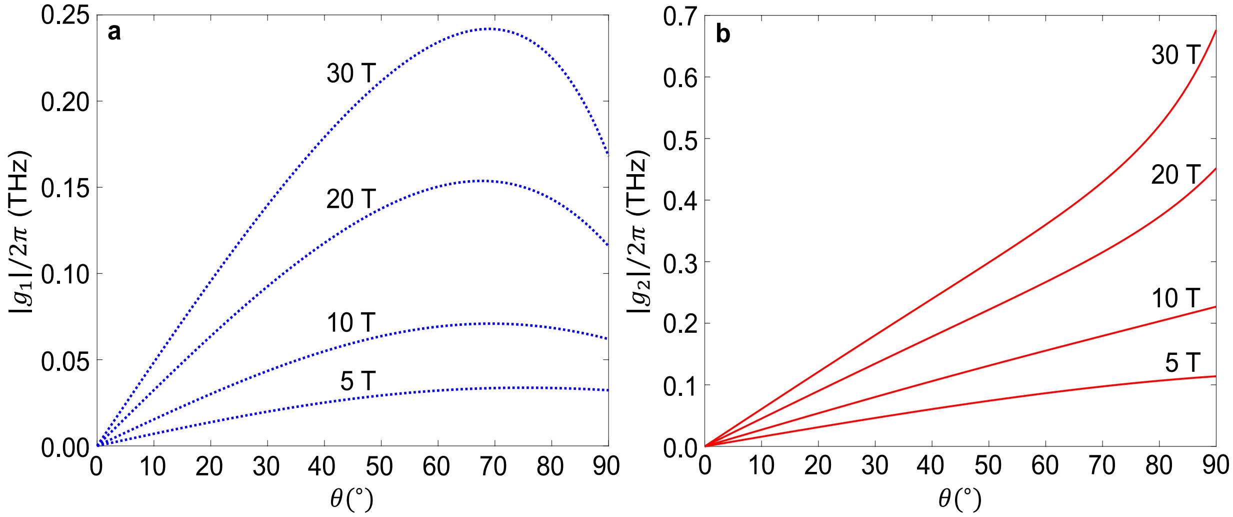

Supplementary Fig. 13. Coupling strength variation with θ. **a,** Plots of $|g_1|/2\pi$ vs. θ for different fixed magnetic fields, and similarly for $|g_2|/2\pi$ vs. θ in **b**. Importantly, we observe that $|g_2|/2\pi$ is a monotonically increasing function of θ.

References

1. Noe, G. T. *et al.* Single-shot terahertz time-domain spectroscopy in pulsed high magnetic fields. *Optics express* **24**, 30328–30337 (2016).

2. Herrmann, G. F. Resonance and high frequency susceptibility in canted antiferromagnetic substances. *Journal of Physics and Chemistry of Solids* **24**, 597–606 (1963).

3. Hahn, S. E. *et al.* Inelastic neutron scattering studies of yfeo 3. *Physical Review B* **89**, 014420 (2014).

4. Koshizuka, N. & Hayashi, K. Raman scattering from magnon excitations in rfeo3. *Journal of the Physical Society of Japan* **57**, 4418–4428 (1988).

5. Amelin, K. *et al.* Terahertz absorption spectroscopy study of spin waves in orthoferrite yfeo 3 in a magnetic field. *Physical Review B* **98**, 174417 (2018).
